# Major depressive disorder and cardiometabolic diseases: a bidirectional Mendelian randomisation study

**DOI:** 10.1007/s00125-020-05131-6

**Published:** 2020-04-08

**Authors:** Bowen Tang, Shuai Yuan, Ying Xiong, Qiqiang He, Susanna C. Larsson

**Affiliations:** 1 Department of Global Public Health, Karolinska Institutet, Stockholm, Sweden; 2 Unit of Cardiovascular and Nutritional Epidemiology, Institute of Environmental Medicine, Karolinska Institutet, Stockholm, Sweden; 3 Department of Surgical Sciences, Uppsala University, Epihubben, Dag Hammarskjölds väg 14 B, 75185 Uppsala, Sweden; 4 Department of Nutrition and Food Hygiene, School of Health Sciences, Wuhan University, Wuhan, China

**Keywords:** Coronary artery disease, Heart failure, Major depression disorder, Mendelian randomisation analysis, Type 2 diabetes

## Abstract

**Aims/hypothesis:**

Observational studies have shown a bidirectional association between major depressive disorder (MDD) and cardiometabolic diseases. We conducted a two-sample bidirectional Mendelian randomisation (MR) study to assess the causal associations of MDD with type 2 diabetes, coronary artery disease (CAD) and heart failure and vice versa.

**Methods:**

We extracted summary-level data for MDD, type 2 diabetes, CAD and heart failure from corresponding published large genome-wide association studies of individuals mainly of European-descent. In total, 96 SNPs for MDD, 202 SNPs for type 2 diabetes, 44 SNPs for CAD and 12 SNPs for heart failure were proposed as instrumental variables at the genome-wide significance level (*p* < 5 × 10^−8^). The random-effects inverse-variance weighted method was used for the main analyses.

**Results:**

Genetic liability to MDD was significantly associated with type 2 diabetes and CAD at the Bonferroni-corrected significance level. The ORs of type 2 diabetes and CAD were respectively 1.26 (95% CI 1.10, 1.43; *p* = 6 × 10^−4^) and 1.16 (95% CI 1.05, 1.29; *p* = 0.0047) per one-unit increase in log_*e*_ odds of MDD. There was a suggestive association between MDD and heart failure (OR 1.11 [95% CI 1.01, 1.21]; *p* = 0.033). We found limited evidence supporting causal effects of cardiometabolic diseases on MDD risk in the reverse MR analyses.

**Conclusions/interpretation:**

The present study strengthened the evidence that MDD is a potential risk factor for type 2 diabetes and CAD. Whether MDD is causally related to heart failure needs further study.

**Data availability:**

All data included in this study were uploaded as supplements and are also publicly available through published GWASs and open GWAS datasets (UK Biobank, 23andMe and Psychiatric Genomics: https://datashare.is.ed.ac.uk/handle/10283/3203; DIAGRAM: http://diagram-consortium.org/downloads.html; CARDIoGRAMplusCD4: www.cardiogramplusc4d.org/; HERMES: http://www.kp4cd.org/datasets/mi).

**Figure:**
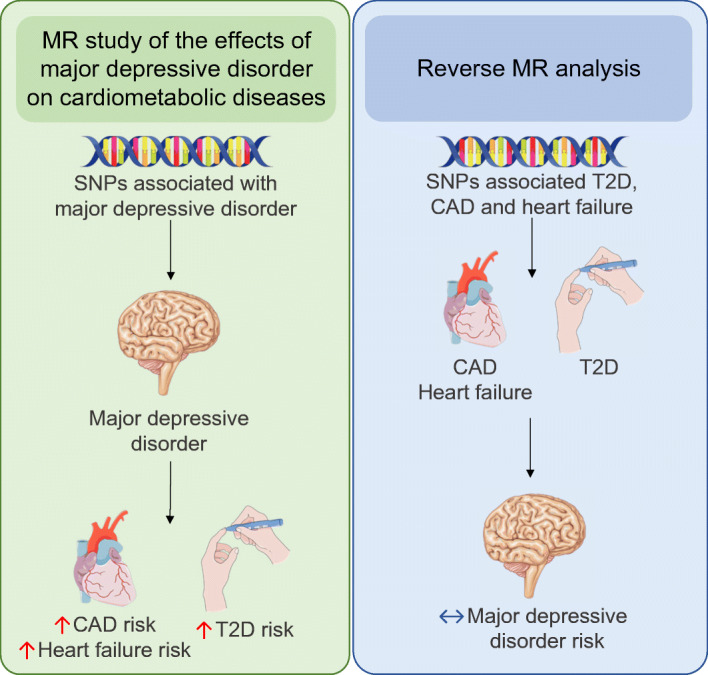
Graphical abstract

**Electronic supplementary material:**

The online version of this article (10.1007/s00125-020-05131-6) contains peer-reviewed but unedited supplementary material, which is available to authorised users.

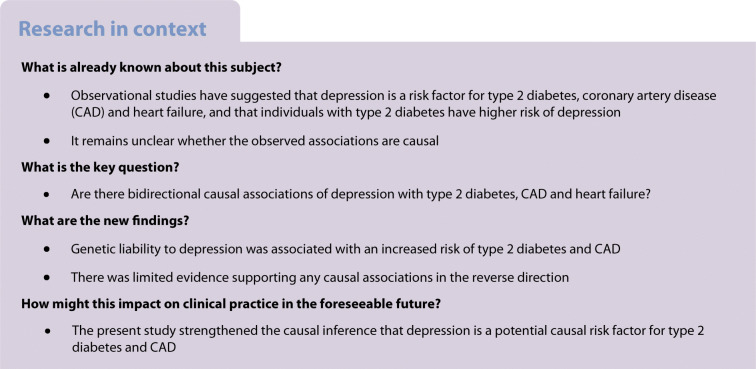



## Introduction

Major depressive disorder (MDD) and type 2 diabetes are both important public health issues globally. It is estimated that around 322 million people had MDD and more than 425 million adults were living with diabetes worldwide in 2015 [1, 2]. Epidemiological data showed that the prevalence of MDD among individuals with type 2 diabetes was twice that in those without diabetes [3], which indicated a potential link between these two prevailing diseases.

Observational studies have reported that MDD is associated with an increased risk of type 2 diabetes through several biological alterations and unhealthy behaviours [4, 5]. It has further been suggested that type 2 diabetes may play a role in the development of MDD, possibly by causing disability and comorbidity [6]. However, whether the mutual association between MDD and type 2 diabetes is causal remains unclear due to potential residual confounding and reverse causation bias in observational studies [7].

Mendelian randomisation (MR) is a method for assessing causal inference of an exposure on an outcome by using genetic variants as instrumental variables for the exposure [8]. This technique diminishes residual confounding because genetic variants are randomly assorted at conception, thereby having no connection to self-selected lifestyle factors, behaviours and environmental factors. In addition, it overcomes reverse causality because genetic variants are fixed regardless of the development or progression of the disease (except for certain cancers).

MDD has been proposed as a risk factor also for other cardiometabolic diseases, such as coronary artery disease (CAD) and heart failure, in observational studies [9–12]. Therefore, we conducted a bidirectional two-sample MR study to explore the causal associations of MDD with major cardiometabolic diseases as well as the causal role of cardiometabolic diseases for MDD.

## Methods

### Study design overview

**Fig. 1:**
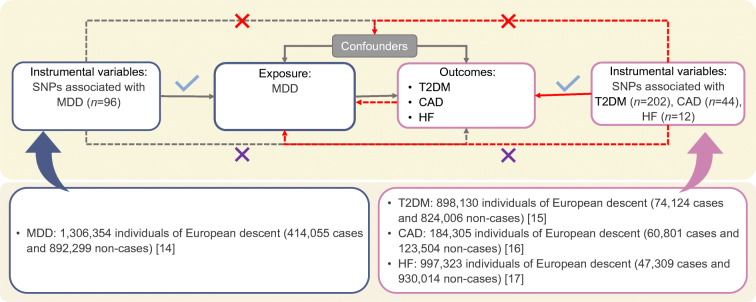
Assumptions and study design of the present bidirectional MR study of the associations of MDD with type 2 diabetes, CAD and heart failure. Grey lines show the relationship across instrumental variables, exposure, and outcomes in the MR study examining the effects of MDD on cardiometabolic diseases, and red lines show these relationships in the reverse MR study. Solid lines represent relationship that were observed, whereas dashed lines represent associations that would violate the MR assumptions (i.e. relationships that are not allowed/did not exist in the present MR study). Blue ticks indicate that genetic variants are associated with the exposure/outcome; red crosses indicate that genetic variants are not associated with any confounders of the exposure–outcome association; purple crosses indicate that genetic variants exert effects on the outcome only via the exposure. HF, heart failure; T2DM, type 2 diabetes

A brief description of the bidirectional MR design is displayed in Fig. 1. There are three key assumptions for MR: (1) genetic variants associate with the exposure of interest; (2) genetic variants are not associated with any confounders of the exposure–outcome association; and (3) genetic variants exert effects on the outcome only via the exposure [13]. We used summary-level data from meta-analyses of genome-wide association studies (GWASs) of MDD, type 2 diabetes, CAD and heart failure. Details of the data sources utilised in the present study are summarised in electronic supplementary material (ESM) Table 1. Studies included in the original GWASs had been approved by a relevant institutional review board and the present MR study has been approved by the Swedish Ethical Review Authority.

### Data sources and SNP selection for MDD

We used the hitherto largest published GWAS for MDD that was conducted in a population of European-descent [14], which included 807,553 individuals (246,363 with MDD and 561,190 without MDD) in the discovery stage and 1,306,354 individuals (414,055 with MDD and 892,299 without MDD) in the replication stage [14]. In total, 102 independent variants were identified, of which 96 reached genome-wide significance (*p* < 5 × 10^−8^) and were selected as instrumental variables for MDD. Summary-level data from the UK Biobank and Psychiatric Genomics Consortium, including 500,199 individuals (170,756 with MDD and 329,443 without MDD), were publicly available and used in the reverse-direction MR analysis.

### Data sources and SNP selection for cardiometabolic diseases

Summary-level data for type 2 diabetes were available from a meta-analysis of 32 GWASs, including 898,130 individuals of European-descent (74,124 with type 2 diabetes and 824,006 without type 2 diabetes) [15]. We selected the 202 SNPs associated with type 2 diabetes at the genome-wide significant level (*p* < 5 × 10^−8^) in the model without adjustment for BMI out of 403 distinct association signals. All 403 SNPs, and SNPs associated with type 2 diabetes at *p* < 5 × 10^−8^ in the BMI-adjusted model, were used in sensitivity analyses.

Summary-level data for CAD were obtained from an 1000 genomes-based GWAS meta-analysis of 48 studies involving 60,801 individuals with CAD and 123,504 without CAD (77% of participants were of European ancestry) [16]. Fifty-eight distinct loci associated with CAD were discovered in this GWAS, of which 44 SNPs reached genome-wide significance and were used as instrumental variables for CAD in the reverse-direction MR analysis.

Summary-level data for heart failure were extracted from a GWAS of 47,309 individuals with heart failure and 930,014 individuals without heart failure from the Heart Failure Molecular Epidemiology for Therapeutic Targets (HERMES) Consortium [17]. Twelve SNPs were reported to be associated with heart failure at the genome-wide significance level. We included all 12 SNPs in the reverse-direction MR analysis.

Details of the SNPs related to MDD and their associations with cardiometabolic diseases are shown in ESM Tables 2–4. SNPs related to cardiometabolic diseases and the associations with MDD are shown in ESM Tables 5–7.

### Statistical analyses

We calculated a Wald ratio estimate for each genetic variant and summarised the estimates using the random-effects inverse-variance weighted (IVW) method. The IVW method with random effects provides a concise estimation and takes into account potential heterogeneity among the Wald ratio estimates from individual SNPs [18]. Furthermore, we conducted several sensitivity analyses by using the weighted median, MR-Egger regression and MR pleiotropy residual sum and outlier (MR-PRESSO) methods as well as leave-one-out analysis. ORs and CIs were scaled to one-unit increment of log_*e*_ OR of MDD, type 2 diabetes, CAD and heart failure.

The power calculation was based on the results of an online tool using several parameters, including sample size of the outcome GWAS, variance explained by selected SNPs and expected effect size [19]. The ‘get-or-from-lor’ commend in the TwoSampleMR package was used to estimate the explained variance for binary traits. The SNPs that were unavailable in the outcome datasets were replaced by proxies at *R*^2^ > 0.90. SNPs with a minor allele frequency above 0.42 were considered to be strand-ambiguous and removed from the analysis. The Bonferroni method was used to correct for multiple testing and, therefore, we considered associations with *p* values below 0.0083 (0.05/6) as strong evidence of associations. Results with *p* values between 0.0083 and 0.05 were regarded as suggestive associations. All analyses were two-sided and conducted using the TwoSampleMR package (https://mrcieu.github.io/TwoSampleMR/) in R software (version 3.6.0; www.r-project.org/).

## Results

### Causal effect of MDD on cardiometabolic diseases

Among the 96 MDD-associated SNPs, six were not available in the dataset of type 2 diabetes, and five of the six unavailable SNPs were replaced by suitable proxy SNPs. Furthermore, six SNPs were excluded due to ambiguous palindrome, resulting in 89 SNPs as instrumental variables in the analysis of type 2 diabetes and 90 SNPs in the analyses of CAD and heart failure (ESM Tables 2–4). The statistical power to detect an OR of 1.2 was 80% in the analysis of type 2 diabetes but only 55% and 63%, respectively, in the analyses of CAD and heart failure (ESM Table 8).

**Fig. 2:**
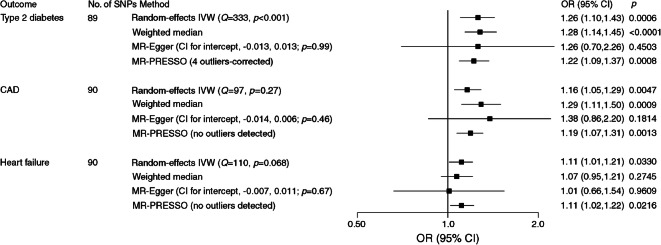
Associations of genetic liability to MDD with risk of type 2 diabetes, CAD and heart failure

Genetic liability to MDD was significantly associated with type 2 diabetes and CAD risk and was suggestively associated with heart failure risk (Fig. 2). The OR of type 2 diabetes per one-unit increase in log_*e*_ odds of MDD liability was 1.26 (95% CI 1.10, 1.43; *p* = 6 × 10^−4^) in the random-effects IVW model. The association was consistent across sensitivity analyses, albeit non-significant in the MR-Egger regression analysis. The MR-PRESSO analysis detected four outliers and provided an OR of type 2 diabetes of 1.22 (95% CI 1.09, 1.37; *p* = 8 × 10^−4^) after outlier correction. The Cochran’s *Q* value implied a substantial heterogeneity among estimates obtained from individual SNPs (*Q* = 333, *p* < 0.001) but the MR-Egger regression analysis yielded no indication of potential non-balanced pleiotropy (CI for intercept, −0.013, 0.013; *p* = 0.99). Moreover, leave-one-out analysis indicated that the observed association was not driven by any single SNP (ESM Fig. 1). The association was slightly weaker and borderline significant in a sensitivity analysis using SNPs associated with type 2 diabetes in the BMI-adjusted model (OR 1.15 [95% CI 1.03, 1.30]; *p* = 0.0166) (ESM Fig. 2).

The ORs per one-unit increase in log_*e*_ odds of MDD liability were 1.16 (95% CI 1.05, 1.29; *p* = 0.005) for CAD and 1.11 (95% CI 1.01, 1.21; *p* = 0.033) for heart failure (Fig. 2). There was no observed heterogeneity (*p* of Cochran’s *Q* > 0.05), no pleiotropy (*p* for intercept, >0.05) and no outliers in the analysis of CAD or heart failure. Leave-one-out analysis indicated that the association with CAD was not driven by any single SNP (ESM Fig. 3) but six outlier SNPs were identified in the analysis of heart failure (ESM Fig. 4). We proceeded and searched for traits associated with those six outliers in a GWAS catalogue but found no likely trait that could confound the observed association between MDD and heart failure (ESM Table 4).

### Causal effect of cardiometabolic diseases on MDD

**Fig. 3:**
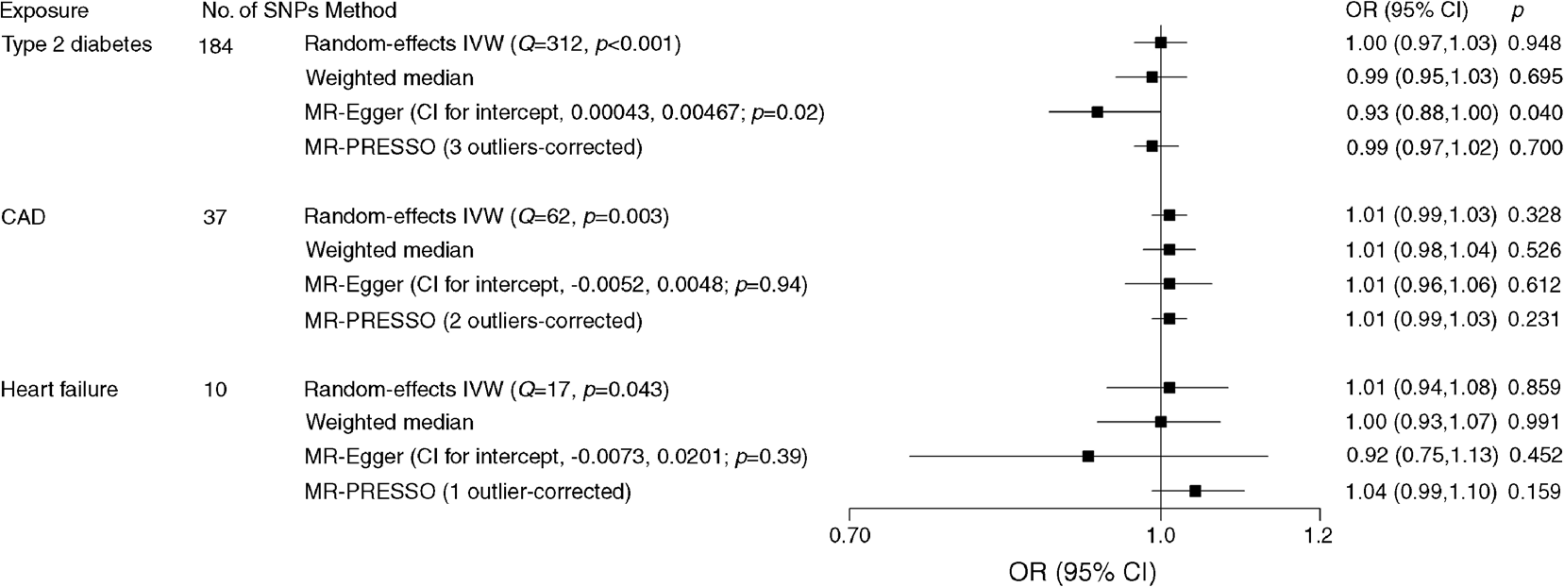
Associations of genetic liability to type 2 diabetes, CAD and heart failure with MDD

After palindromic SNP exclusion and proxy replacement, we used 184 SNPs for type 2 diabetes, 37 SNPs for CAD and ten SNPs for heart failure as instrumental variables in the analysis of the effects of cardiometabolic diseases on MDD (ESM Tables 5–7). We had high power (over 90% power to detect an OR of 1.1) to detect weak associations of type 2 diabetes and CAD with MDD, but lower power in the analysis of the effect of heart failure on MDD (87% power to detect an OR of 1.20) (ESM Table 8). None of the three cardiometabolic diseases were associated with MDD, with ORs close to 1 (Fig. 3). Although there was suggestive evidence of an association between type 2 diabetes and MDD in the MR-Egger analysis (OR 0.93 [95% CI 0.88, 1.00]; *p* = 0.040), no association was observed after correction for three outliers (OR 0.99 [95% CI 0.97, 1.02]; *p* = 0.700) in the MR-PRESSO analysis (Fig. 3). Furthermore, there was no association between type 2 diabetes and MDD in sensitivity analyses using all 403 SNPs or using the 172 SNPs from the BMI-adjusted model (ESM Fig. 5).

## Discussion

In the present bidirectional two-sample MR study, we observed a significant positive association of genetic liability to MDD with type 2 diabetes and CAD, and a suggestive association with heart failure. The reverse MR analysis provided no evidence that liability to type 2 diabetes, CAD or heart failure was related to MDD.

Adverse effects of MDD on type 2 diabetes development have been demonstrated consistently in observational studies. A meta-analysis of nine prospective studies proposed that depression was a risk factor for the onset of type 2 diabetes and reported a pooled RR of 1.26 (95% CI 1.13, 1.39), which is in agreement with our findings [20]. Results of another updated meta-analysis, which included 13 prospective studies with 6916 incident cases of type 2 diabetes, showed that having depression increased the risk of type 2 diabetes by 60% [21]. Additionally, a positive association between MDD and type 2 diabetes was supported by three large-scale cohort studies with medium or long follow-up periods (individual studies included 65,381 women followed up for 10 years, 11,694 adults for 6 years, and 5201 adults for 3.2 years) [22–24]. In contrast, epidemiological data on the effect of type 2 diabetes on MDD risk are inconclusive [25]. A systematic review including 83 studies found that type 2 diabetes could not independently predict MDD risk [26], but some recently published studies reported a significant positive association in both directions [21–24]. The present MR study found no evidence supporting a causal detrimental effect of type 2 diabetes on MDD. A cohort study that followed 65,381 women for 10 years found that the risk of developing clinical depression was higher in diabetic women, particularly in those who received insulin therapy, compared with non-diabetic women [22]. Another cohort study reported that treated but not untreated type 2 diabetes was associated with an increased risk of depression [23]. These studies suggest that MDD risk in type 2 diabetes may differ depending on type 2 diabetes treatment [22, 23].

We found evidence that MDD may be causally associated with risk of CAD and possibly heart failure, thereby confirming the results of observational studies [9–12, 27]. Notably, diabetes and CAD have been proposed as risk factors for heart failure, and CAD can explain more than 60% of heart failure cases [28]. Thus, the suggestive association between MDD and heart failure might be partly mediated via type 2 diabetes and CAD.

There are several possible mechanisms that can explain the causal effect of MDD on type 2 diabetes risk. A series of biological abnormalities related to depression, including increased counter-regulatory hormone release and activity, alterations in glucose transport function and increased immunoinflammatory activation, may influence the risk of type 2 diabetes [5]. In addition, lifestyle factors, such as smoking and alcohol consumption, may play a mediating role in the pathway from depression to type 2 diabetes [29].

In traditional observational studies of the effect of depression on type 2 diabetes, the results are prone to be biased by reverse causality, as impaired glucose tolerance may lead to depression before diabetes symptoms manifest. Antidepressant medication, which has been demonstrated to be associated with hyperglycaemia, may introduce residual confounding [25]. We used the MR study design, thereby avoiding reverse causality and minimising residual confounding. Another strength is that we extracted summary-level data from the hitherto largest GWAS for MDD and type 2 diabetes and, therefore, we had high power to detect even weak associations. A limitation of the present study is the potential of pleiotropy. However, we detected no directional pleiotropy in the MR-Egger regression analysis and the estimates were consistent when using the weighted median and MR-PRESSO analyses, which indicated a negligible distortion by potential pleiotropy. A previous MR study revealed that BMI was a risk factor for both MDD [30] and type 2 diabetes [31], indicating that BMI might be a pleiotropic factor or confounder in the pathway from MDD to type 2 diabetes. However, in a sensitivity analysis using BMI-adjusted estimates for the genetic associations with type 2 diabetes, the effect of MDD on type 2 diabetes remained at the conventional level of significance (*p* < 0.05). This implies that the causal association between MDD and type 2 diabetes was not completely mediated or biased by BMI. Another limitation is that population stratification may have affected the result for the association between CAD and MDD because the GWAS of CAD included participants of different ancestry. However, as the majority (77%) of individuals were of European ancestry, any population stratification bias was expected to be small.

The present MR study strengthens the evidence that MDD is a potential risk factor for type 2 diabetes and CAD. However, there was no genetic support for a causal effect of type 2 diabetes or CAD on MDD. Whether MDD is causally related to heart failure needs further investigation. Given the high disease burden related to the causal link, it is recommended that MDD prevention, management and treatment should be enhanced for type 2 diabetes prevention.

## Electronic supplementary material

ESM 1 (PDF 1770 kb)

## Data Availability

All data included in this study were uploaded as supplements and are also publicly available through published GWASs and open GWAS datasets (UK Biobank, 23andMe and Psychiatric Genomics: https://datashare.is.ed.ac.uk/handle/10283/3203; DIAGRAM: http://diagram-consortium.org/downloads.html; CARDIoGRAMplusCD4: www.cardiogramplusc4d.org/; HERMES: http://www.kp4cd.org/datasets/mi).

## Abbreviations

CAD: Coronary artery disease
GWAS: Genome-wide association study
IVW: Inverse-variance weighted
MDD: Major depressive disorder
MR: Mendelian randomisation
MR-PRESSO: Mendelian randomisation pleiotropy residual sum and outlier
