# Supplementary material for: Major depressive disorder and cardiometabolic diseases: a bidirectional Mendelian randomisation study
Source: Diabetologia. 2020 Apr 8;63(7):1305–11. doi: 10.1007/s00125-020-05131-6 (PMC7286869; doi:10.1007/s00125-020-05131-6)
Supplement: Supplementary file 1 — (PDF 1770 kb) [file 125_2020_5131_MOESM1_ESM.pdf]

ESM Table 1. Details of studies and datasets used for analyses

Exposure/Outcome	Consortium or cohort study	Participants	Web source if publicly available
Major depressive disorder	UK Biobank, 23andMe and Psychiatric Genomics Consortium.	807,553 individuals (246,363 MDD cases and 561,190 controls) in the discovery stage and 1,306,354 individuals (414,055 cases and 892,299 controls) in the replication stage of European ancestry	https://datashare.is.ed.ac.uk/handle/10283/3203
Type 2 diabetes	DIAGRAM consortium	89,8130 individuals (74,124 type 2 diabetes cases and 824,006 controls) of European ancestry	http://diagram-consortium.org/downloads.html
Coronary artery disease	CARDIoGRAMplusC4D consortium	184,305 individuals (60,801coronary artery disease cases and 123,504 non-cases) of mainly European (77%) and Asian (19%) ancestry	www.cardiogramplusc4d.org/
Heart failure	Heart Failure Molecular Epidemiology for Therapeutic Targets (HERMES) Consortium	977,323 individuals (47,309 heart failure cases and 930,014 non-cases) of European ancestry	http://www.kp4cd.org/datasets/mi

ESM Table 2. Characteristics of the single-nucleotide polymorphisms associated with major depressive disorder and their associations with type 2 diabetes

		Major depressive disorder						Type 2 diabetes								
Chr	SNP	EA	NEA	EAF	Beta	SE	P	EA	NEA	EAF	Beta[a]	SE	P	Proxy[#]	R^2	Ambiguous palindrome*
1	rs1002656	T	C	0.70	-0.021	0.002	4.84E-19	T	C	0.71	-0.005	0.007	0.490			
5	rs10061069	C	G	0.22	-0.025	0.003	5.17E-22	C	G	0.21	-0.007	0.008	0.370			
14	rs10149470	A	G	0.49	-0.021	0.002	6.88E-22	A	G	0.49	-0.008	0.006	0.230			
3	rs4346585	T(C)	C(T)	0.70	-0.016	0.002	4.87E-12	C	T	0.68	0.009	0.007	0.200	rs10212298	1.0	
10	rs1021363	A	G	0.35	0.023	0.002	3.77E-23	A	G	0.35	0.006	0.007	0.350			
14	rs1045430	T	G	0.48	-0.023	0.002	4.44E-25	T	G	0.48	-0.005	0.006	0.400			
1	rs10789214	T	C	0.57	0.014	0.002	3.81E-10	T	C	0.56	0.021	0.006	0.002			
9	rs1982277	T(G)	C(C)	0.76	0.021	0.003	1.28E-15	G	C	0.74	0.021	0.007	0.003	rs10809535	1.0	
9	rs10817969	T	G	0.72	0.017	0.002	1.32E-12	T	G	0.72	0.003	0.007	0.690			
1	rs10890020	A	G	0.52	-0.027	0.002	3.31E-34	A	G	0.51	0.004	0.006	0.540			
1	rs10913112	T	C	0.38	-0.021	0.002	1.72E-20	T	C	0.38	0.004	0.007	0.590			
3	rs1095626	T	C	0.58	-0.029	0.002	6.31E-39	T	C	0.58	0.018	0.006	0.006			
5	rs11135349	A	C	0.47	-0.025	0.002	2.18E-30	A	C	0.46	0.013	0.006	0.049			
1	rs113188507	A	G	0.28	0.023	0.003	2.94E-21	A	G	0.28	0.011	0.007	0.086			
14	rs1152578	T	C	0.44	-0.015	0.002	4.19E-12	T	C	0.44	0.008	0.006	0.220			
1	rs11579246	A	G	0.91	0.032	0.004	9.34E-18	A	G	0.90	0.014	0.011	0.190			
2	rs12052908	A	T	0.53	-0.021	0.002	3.98E-22	A	T	0.52	-0.018	0.006	0.005			Yes

2	rs1226412	T	C	0.79	0.023	0.003	3.35E-18	T	C	0.80	-0.003	0.008	0.720			
20	rs12624433	A	G	0.26	0.019	0.003	2.16E-14	A	G	0.26	0.014	0.007	0.052			
16	rs12923444	A	C	0.56	-0.024	0.002	1.76E-24	A	C	0.56	0.005	0.006	0.400			
18	rs12966052	C	G	0.18	-0.019	0.003	3.76E-11	C	G	0.18	0.013	0.008	0.110			
18	rs12967143	C	G	0.70	-0.026	0.002	1.61E-27	C	G	0.71	0.010	0.007	0.140			
18	rs12967855	A	G	0.33	0.021	0.002	1.32E-19	A	G	0.33	0.013	0.007	0.068			
3	rs13084037	A	G	0.77	-0.022	0.003	1.79E-17	A	G	0.77	0.004	0.008	0.610			
13	rs1343605	A	C	0.38	0.023	0.002	1.62E-25	A	C	0.38	0.013	0.007	0.050			
9	rs1354115	A	C	0.62	0.019	0.002	4.39E-17	A	C	0.63	-0.001	0.007	0.870			
13	rs1409379	T	C	0.76	0.016	0.003	8.26E-11	T	C	0.76	0.016	0.007	0.029			
11	rs1448938	A	G	0.42	0.017	0.002	3.74E-15	A	G	0.42	-0.008	0.006	0.220			
1	rs1466887	T	C	0.55	-0.013	0.002	1.37E-08	T	C	0.55	0.002	0.007	0.760			
2	rs1568452	T	C	0.39	0.029	0.002	3.08E-40	T	C	0.38	0.010	0.007	0.140			
7	rs16887442	T	C	0.43	0.015	0.002	1.25E-11	T	C	0.43	-0.006	0.006	0.360			
1	rs169235	A	G	0.75	-0.017	0.003	1.61E-11	A	G	0.75	-0.015	0.007	0.044			
1	rs17641524	T	C	0.21	-0.024	0.003	7.90E-20	T	C	0.22	0.010	0.008	0.190			
1	rs1890946	T	C	0.47	-0.019	0.002	8.62E-19	T	C	0.47	0.003	0.006	0.640			
6	rs1933802	C	G	0.45	-0.020	0.002	1.36E-19	C	G	0.46	0.006	0.006	0.360			Yes
6	rs9363467	T(C)	C(T)	0.60	0.016	0.002	2.50E-12	C	T	0.61	0.011	0.007	0.084	rs1938082	1.0	
14	rs1956373	T	G	0.74	-0.017	0.003	6.08E-12	T	G	0.75	0.005	0.007	0.460			

11	rs198457	T	C	0.19	-0.021	0.003	5.41E-14	T	C	0.18	0.013	0.009	0.140			
6	rs200949	A	G	0.87	0.043	0.003	2.13E-37	A	G	0.88	-0.014	0.010	0.160			
6	rs2029865	A	T	0.45	-0.018	0.002	4.02E-17	A	T	0.45	-0.011	0.006	0.100			Yes
7	rs2043539	A	G	0.42	0.022	0.002	8.32E-24	A	G	0.41	0.023	0.007	0.000			
7	rs2247523	C	G	0.53	-0.016	0.002	9.05E-14	C	G	0.53	-0.014	0.006	0.032			Yes
11	rs2509805	T	C	0.32	0.019	0.002	4.92E-16	T	C	0.32	-0.008	0.007	0.240			
1	rs2568958	A	G	0.62	0.034	0.002	4.45E-52	A	G	0.61	0.025	0.007	0.000			
9	rs263645	A	T	0.54	0.018	0.002	6.92E-16	A	T	0.54	0.012	0.006	0.067			Yes
9	rs2670139	T	C	0.76	-0.018	0.003	1.84E-12	T	C	0.75	-0.038	0.007	0.000			
6	rs2876520	C	G	0.53	-0.019	0.002	2.65E-17	C	G	0.53	-0.006	0.006	0.350			Yes
1	rs301799	T	C	0.57	-0.025	0.002	4.05E-31	T	C	0.58	-0.002	0.006	0.820			
5	rs30266	A	G	0.33	0.032	0.002	1.99E-45	A	G	0.32	0.024	0.007	0.001			
5	rs3099439	T	C	0.53	-0.021	0.002	1.61E-21	T	C	0.53	0.016	0.006	0.013			
12	rs3213572	A	G	0.47	0.020	0.002	5.51E-20	A	G	0.47	0.032	0.006	0.000			
15	rs34488670	T	C	0.79	-0.019	0.003	4.69E-13	T	C	0.79	-0.011	0.008	0.150			
9	rs34653192	C	G	0.32	-0.020	0.002	3.39E-16	C	G	0.33	-0.007	0.007	0.330			
4	rs34937911	T	C	0.88	0.024	0.003	5.39E-13	T	C	0.88	0.000	0.010	0.990			
3	rs141954845	A(G)	G(A)	0.39	0.018	0.002	1.18E-15	G	A	0.41	-0.009	0.007	0.170	rs35198002	1.0	
4	rs35553410	T	C	0.75	-0.016	0.003	2.18E-10	T	C	0.75	0.012	0.007	0.110			
9	rs3793577	A	G	0.47	-0.021	0.002	5.58E-22	A	G	0.47	0.007	0.006	0.270			
7	rs3823624	T	C	0.81	0.029	0.003	1.61E-26	T	C	0.80	0.030	0.008	0.000			

4	rs45510091	A	G	0.95	0.046	0.005	7.74E-21	A	G	0.95	0.013	0.014	0.350		
13	rs4772087	T	C	0.37	0.021	0.002	2.34E-20	T	C	0.38	0.020	0.007	0.002		
12	rs56314503	T	G	0.75	-0.020	0.003	1.28E-16	T	G	0.76	-0.015	0.007	0.045		
16	rs56887639	A	G	0.73	-0.017	0.002	6.04E-13	A	G	0.73	0.004	0.007	0.630		
11	rs57344483	A	G	0.93	-0.024	0.004	5.26E-09	A	G	0.92	0.012	0.012	0.320		
7	rs58104186	A	G	0.47	0.019	0.002	3.56E-18	A	G	0.46	0.002	0.006	0.820		
11	rs58621819	A	T	0.79	-0.017	0.003	2.17E-10	A	T	0.80	-0.061	0.008	0.000		
22	rs5995992	T	C	0.72	-0.030	0.002	2.60E-35	T	C	0.72	-0.022	0.007	0.002		
5	rs60157091	T	C	0.52	0.021	0.002	5.54E-21	T	C	0.52	-0.010	0.006	0.100		
20	rs143186028	T(C)	G(T)	0.18	0.024	0.003	7.23E-18	C	T	0.19	0.011	0.008	0.160	rs6072350	1.0
11	rs61902811	A	G	0.37	-0.029	0.002	3.57E-39	A	G	0.36	-0.012	0.007	0.062		
14	rs61990288	A	G	0.51	-0.025	0.002	1.96E-31	A	G	0.50	-0.011	0.006	0.095		
18	rs62091461	T	C	0.23	-0.020	0.003	1.75E-14	T	C	0.24	0.013	0.007	0.088		
2	rs62188629	A	G	0.31	0.017	0.002	5.07E-13	A	G	0.32	0.010	0.007	0.160		
3	rs6783233	T	C	0.28	0.018	0.002	1.14E-13	T	C	0.28	0.001	0.007	0.950		
9	rs7030813	T	C	0.37	0.025	0.002	8.88E-30	T	C	0.38	0.018	0.007	0.006		
11	rs7117514	A	G	0.54	-0.017	0.002	3.64E-14	A	G	0.54	0.005	0.006	0.450		
16	rs7193263	A	G	0.67	-0.021	0.002	3.83E-19	A	G	0.67	-0.001	0.007	0.930		
16	rs7198928	T	C	0.62	0.022	0.002	3.71E-23	T	C	0.62	-0.005	0.007	0.490		
16	rs7200826	T	C	0.26	0.027	0.003	3.09E-28	T	C	0.27	0.004	0.007	0.600		
18	rs7227069	A	G	0.43	0.024	0.002	2.13E-28	A	G	0.44	0.001	0.006	0.850		
18	rs7241572	A	G	0.20	0.020	0.003	2.56E-13	A	G	0.20	0.019	0.008	0.020		

6	rs725616	T	C	0.36	0.014	0.002	1.06E-09	T	C	0.36	0.020	0.007	0.003	
1	rs72710803	A	C	0.91	-0.025	0.004	1.35E-10	A	C	0.92	-0.020	0.012	0.090	
17	rs75581564	A	G	0.12	0.025	0.003	3.49E-13	A	G	0.11	0.003	0.010	0.790	
2	rs7585722	T	C	0.85	-0.022	0.003	5.47E-13	T	C	0.85	0.018	0.009	0.047	
4	rs7685686	A	G	0.58	0.017	0.002	5.52E-15	A	G	0.57	0.021	0.007	0.002	
6	rs7758630	A	T	0.41	-0.017	0.002	7.79E-14	A	T	0.40	0.001	0.007	0.900	
7	rs7807677	T	C	0.55	0.021	0.002	5.40E-22	T	C	0.57	0.022	0.006	0.000	
12	rs78337797	T	G	0.88	0.023	0.003	1.37E-11	T	G	0.88	-0.022	0.010	0.025	
8	rs7837935	T	G	0.15	-0.023	0.003	1.15E-13	T	G	0.15	-0.019	0.009	0.036	
11	rs7932640	T	C	0.44	0.023	0.002	3.40E-25	T	C	0.45	0.010	0.006	0.110	
15	rs8037355	T	C	0.56	-0.019	0.002	1.52E-18	T	C	0.55	-0.006	0.006	0.350	
9	rs913930	A	G	0.64	-0.022	0.002	1.03E-21	A	G	0.64	-0.007	0.007	0.290	
13	rs9545360	A	C	0.18	-0.017	0.003	6.70E-09	A	C	0.18	0.013	0.008	0.110	
13	rs9592461	A	G	0.49	0.024	0.002	1.17E-28	A	G	0.48	0.009	0.006	0.170	
10	rs997934	T	C	0.38	0.016	0.002	4.60E-13	T	C	0.38	-0.005	0.007	0.420	
8	rs67436663	G	C	0.24	0.014	0.003	2.83E-08	NA	NA	NA	NA	NA	NA	<0.9

Chr indicates chromosome; SNP, single nucleotide polymorphism; EA, effect allele; NEA, non-effect allele; EAF, effect allele frequency; SE, standard error.

[a] Beta presents natural logarithm of odds ratio.

[#] Genetic variants which were not available in the outcome summary statistics data were replaced by their proxies (R^2>0.90). The effect allele and non-effect allele of proxies were noted in the brackets.

[*] Palindromic SNPs with minor allele frequency of >0.42 were considered to be strand-ambiguous and removed from final analysis.

ESM Table 3. Characteristics of the single-nucleotide polymorphisms associated with major depressive disorder and their associations with coronary artery disease

		Major depressive disorder						Coronary artery disease						
Chr	SNP	EA	NEA	EAF	Beta[a]	SE	P	EA	NEA	EAF	Beta[a]	SE	P	Ambiguous palindrome[*]
1	rs1002656	T	C	0.70	-0.021	0.002	4.84E-19	T	C	0.64	-0.001	0.010	8.85E-01	
5	rs10061069	C	G	0.22	-0.025	0.003	5.17E-22	C	G	0.20	-0.023	0.012	5.99E-02	
14	rs10149470	A	G	0.49	-0.021	0.002	6.88E-22	A	G	0.52	-0.009	0.009	3.43E-01	
10	rs1021363	A	G	0.35	0.023	0.002	3.77E-23	A	G	0.37	0.015	0.010	1.20E-01	
14	rs1045430	T	G	0.48	-0.023	0.002	4.44E-25	T	G	0.51	-0.004	0.009	6.97E-01	
1	rs10789214	T	C	0.57	0.014	0.002	3.81E-10	T	C	0.62	0.002	0.010	8.18E-01	
9	rs10817969	T	G	0.72	0.017	0.002	1.32E-12	T	G	0.73	0.008	0.010	4.29E-01	
1	rs10890020	A	G	0.52	-0.027	0.002	3.31E-34	A	G	0.55	-0.005	0.009	6.23E-01	
1	rs10913112	T	C	0.38	-0.021	0.002	1.72E-20	T	C	0.36	-0.014	0.010	1.54E-01	
3	rs1095626	T	C	0.58	-0.029	0.002	6.31E-39	T	C	0.59	0.000	0.009	9.76E-01	
5	rs11135349	A	C	0.47	-0.025	0.002	2.18E-30	A	C	0.46	0.001	0.009	8.99E-01	
1	rs113188507	A	G	0.28	0.023	0.003	2.94E-21	A	G	0.26	0.008	0.011	4.27E-01	
14	rs1152578	T	C	0.44	-0.015	0.002	4.19E-12	T	C	0.44	0.012	0.009	2.03E-01	
1	rs11579246	A	G	0.91	0.032	0.004	8.07E-18	A	G	0.88	-0.009	0.014	5.12E-01	
2	rs12052908	A	T	0.53	-0.021	0.002	3.98E-22	A	T	0.51	-0.018	0.010	6.46E-02	Yes
2	rs1226412	T	C	0.79	0.023	0.003	3.35E-18	T	C	0.74	-0.005	0.011	6.30E-01	
20	rs12624433	A	G	0.26	0.019	0.003	2.16E-14	A	G	0.25	0.006	0.011	5.54E-01	
16	rs12923444	A	C	0.56	-0.024	0.002	1.76E-24	A	C	0.60	0.004	0.010	7.08E-01	
18	rs12966052	C	G	0.18	-0.019	0.003	3.76E-11	C	G	0.17	0.000	0.013	9.74E-01	
18	rs12967143	C	G	0.70	-0.026	0.002	1.61E-27	C	G	0.67	-0.007	0.010	4.91E-01	
18	rs12967855	A	G	0.33	0.021	0.002	1.32E-19	A	G	0.37	-0.010	0.010	3.25E-01	
3	rs13084037	A	G	0.77	-0.022	0.003	1.79E-17	A	G	0.79	-0.010	0.012	4.09E-01	
13	rs1343605	A	C	0.38	0.023	0.002	1.62E-25	A	C	0.38	0.009	0.010	3.57E-01	
9	rs1354115	A	C	0.62	0.019	0.002	4.39E-17	A	C	0.64	0.018	0.010	7.35E-02	

13	rs1409379	T	C	0.76	0.016	0.003	8.26E-11	T	C	0.72	-0.001	0.010	9.57E-01	
3	rs141954845	A	G	0.39	0.018	0.002	1.18E-15	A	G	0.37	-0.009	0.010	3.68E-01	
20	rs143186028	T	G	0.18	0.024	0.003	7.23E-18	T	G	0.15	-0.012	0.013	3.90E-01	
11	rs1448938	A	G	0.42	0.017	0.002	3.74E-15	A	G	0.45	0.006	0.009	4.91E-01	
1	rs1466887	T	C	0.55	-0.013	0.002	1.37E-08	T	C	0.55	0.005	0.010	6.37E-01	
2	rs1568452	T	C	0.39	0.029	0.002	3.08E-40	T	C	0.37	0.013	0.010	1.88E-01	
7	rs16887442	T	C	0.43	0.015	0.002	1.25E-11	T	C	0.46	-0.008	0.009	3.92E-01	
1	rs169235	A	G	0.75	-0.017	0.003	1.61E-11	A	G	0.75	-0.005	0.011	6.15E-01	
1	rs17641524	T	C	0.21	-0.024	0.003	7.90E-20	T	C	0.20	0.020	0.012	8.62E-02	
1	rs1890946	T	C	0.47	-0.019	0.002	8.62E-19	T	C	0.48	0.008	0.010	4.19E-01	
6	rs1933802	C	G	0.45	-0.020	0.002	1.36E-19	C	G	0.46	-0.012	0.009	2.06E-01	Yes
14	rs1956373	T	G	0.74	-0.017	0.003	6.08E-12	T	G	0.72	0.012	0.010	2.44E-01	
9	rs1982277	T	C	0.76	0.021	0.003	1.28E-15	T	C	0.79	0.007	0.012	5.53E-01	
11	rs198457	T	C	0.19	-0.021	0.003	5.41E-14	T	C	0.17	0.001	0.014	9.58E-01	
6	rs200949	A	G	0.87	0.043	0.003	2.13E-37	A	G	0.88	-0.003	0.015	8.34E-01	
6	rs2029865	A	T	0.45	-0.018	0.002	4.02E-17	A	T	0.43	-0.013	0.009	1.52E-01	Yes
7	rs2043539	A	G	0.42	0.022	0.002	8.32E-24	A	G	0.45	0.027	0.009	3.55E-03	
7	rs2247523	C	G	0.53	-0.016	0.002	9.05E-14	C	G	0.52	-0.006	0.009	5.05E-01	Yes
11	rs2509805	T	C	0.32	0.019	0.002	4.92E-16	T	C	0.36	-0.011	0.010	2.84E-01	
1	rs2568958	A	G	0.62	0.034	0.002	4.45E-52	A	G	0.61	0.011	0.010	2.72E-01	
9	rs263645	A	T	0.54	0.018	0.002	6.92E-16	A	T	0.53	0.010	0.009	2.75E-01	Yes
9	rs2670139	T	C	0.76	-0.018	0.003	2.19E-12	T	C	0.72	-0.015	0.010	1.60E-01	
6	rs2876520	C	G	0.53	-0.019	0.002	2.65E-17	C	G	0.54	0.004	0.010	6.65E-01	Yes
1	rs301799	T	C	0.57	-0.025	0.002	4.05E-31	T	C	0.55	0.016	0.009	9.27E-02	
5	rs30266	A	G	0.33	0.032	0.002	1.99E-45	A	G	0.32	0.007	0.010	4.91E-01	
5	rs3099439	T	C	0.53	-0.021	0.002	1.61E-21	T	C	0.51	0.017	0.010	8.24E-02	
12	rs3213572	A	G	0.47	0.020	0.002	5.51E-20	A	G	0.47	0.021	0.009	2.26E-02	
15	rs34488670	T	C	0.79	-0.019	0.003	4.69E-13	T	C	0.81	0.012	0.012	3.26E-01	
9	rs34653192	C	G	0.32	-0.020	0.002	3.39E-16	C	G	0.27	-0.015	0.011	1.95E-01	
4	rs34937911	T	C	0.88	0.024	0.003	5.39E-13	T	C	0.89	0.025	0.015	9.75E-02	

4	rs35553410	T	C	0.75	-0.016	0.003	2.18E-10	T	C	0.77	0.008	0.011	4.51E-01
9	rs3793577	A	G	0.47	-0.021	0.002	5.58E-22	A	G	0.48	-0.007	0.009	4.36E-01
7	rs3823624	T	C	0.81	0.029	0.003	1.61E-26	T	C	0.79	0.023	0.011	4.54E-02
3	rs4346585	T	C	0.70	-0.016	0.002	4.87E-12	T	C	0.75	-0.019	0.012	1.06E-01
4	rs45510091	A	G	0.95	0.046	0.005	7.74E-21	A	G	0.96	-0.020	0.025	4.32E-01
13	rs4772087	T	C	0.37	0.021	0.002	2.34E-20	T	C	0.35	-0.007	0.010	4.65E-01
12	rs56314503	T	G	0.75	-0.020	0.003	1.28E-16	T	G	0.76	-0.016	0.011	1.49E-01
16	rs56887639	A	G	0.73	-0.017	0.002	6.04E-13	A	G	0.73	-0.010	0.010	3.60E-01
11	rs57344483	A	G	0.93	-0.024	0.004	5.26E-09	A	G	0.90	-0.016	0.016	3.05E-01
7	rs58104186	A	G	0.47	0.019	0.002	3.56E-18	A	G	0.48	0.011	0.009	2.25E-01
11	rs58621819	A	T	0.79	-0.017	0.003	2.17E-10	A	T	0.82	0.001	0.013	9.41E-01
22	rs5995992	T	C	0.72	-0.030	0.002	2.60E-35	T	C	0.73	0.009	0.010	3.74E-01
5	rs60157091	T	C	0.52	0.021	0.002	5.54E-21	T	C	0.51	-0.013	0.010	1.75E-01
11	rs61902811	A	G	0.37	-0.029	0.002	5.17E-39	A	G	0.32	-0.011	0.010	2.90E-01
14	rs61990288	A	G	0.51	-0.025	0.002	1.96E-31	A	G	0.44	-0.012	0.010	2.09E-01
18	rs62091461	T	C	0.23	-0.020	0.003	1.75E-14	T	C	0.24	-0.010	0.011	3.55E-01
2	rs62188629	A	G	0.31	0.017	0.002	6.15E-13	A	G	0.30	-0.010	0.011	3.43E-01
8	rs67436663	C	G	0.24	-0.014	0.003	2.83E-08	C	G	0.26	-0.008	0.011	4.71E-01
3	rs6783233	T	C	0.28	0.018	0.002	1.14E-13	T	C	0.26	-0.003	0.011	7.50E-01
9	rs7030813	T	C	0.37	0.025	0.002	8.88E-30	T	C	0.39	0.008	0.010	4.17E-01
11	rs7117514	A	G	0.54	-0.017	0.002	3.64E-14	A	G	0.58	-0.002	0.009	8.47E-01
16	rs7193263	A	G	0.67	-0.021	0.002	3.83E-19	A	G	0.63	-0.010	0.010	3.34E-01
16	rs7198928	T	C	0.62	0.022	0.002	3.71E-23	T	C	0.63	0.001	0.010	8.91E-01
16	rs7200826	T	C	0.26	0.027	0.003	3.09E-28	T	C	0.24	0.020	0.011	6.25E-02
18	rs7227069	A	G	0.43	0.024	0.002	2.13E-28	A	G	0.37	0.007	0.010	4.76E-01
18	rs7241572	A	G	0.20	0.020	0.003	2.56E-13	A	G	0.18	-0.003	0.013	7.92E-01
6	rs725616	T	C	0.36	0.014	0.002	1.06E-09	T	C	0.35	0.001	0.010	9.02E-01
1	rs72710803	A	C	0.91	-0.025	0.004	1.35E-10	A	C	0.91	-0.010	0.016	5.39E-01
17	rs75581564	A	G	0.12	0.025	0.003	3.49E-13	A	G	0.10	-0.007	0.017	6.61E-01
2	rs7585722	T	C	0.85	-0.022	0.003	5.47E-13	T	C	0.85	0.027	0.014	6.17E-02

4	rs7685686	A	G	0.58	0.017	0.002	5.52E-15	A	G	0.59	0.010	0.009	2.71E-01
6	rs7758630	A	T	0.41	-0.017	0.002	7.79E-14	A	T	0.37	0.008	0.010	4.28E-01
7	rs7807677	T	C	0.55	0.021	0.002	5.40E-22	T	C	0.56	0.020	0.009	3.35E-02
12	rs78337797	T	G	0.88	0.023	0.003	1.37E-11	T	G	0.89	-0.002	0.017	9.19E-01
8	rs7837935	T	G	0.15	-0.023	0.003	1.15E-13	T	G	0.18	-0.015	0.012	2.25E-01
11	rs7932640	T	C	0.44	0.023	0.002	3.40E-25	T	C	0.43	-0.006	0.009	5.49E-01
15	rs8037355	T	C	0.56	-0.019	0.002	1.52E-18	T	C	0.55	0.011	0.009	2.43E-01
9	rs913930	A	G	0.64	-0.022	0.002	1.03E-21	A	G	0.65	-0.008	0.010	4.28E-01
6	rs9363467	T	C	0.60	0.016	0.002	2.50E-12	T	C	0.61	0.009	0.010	3.31E-01
13	rs9545360	A	C	0.18	-0.017	0.003	6.70E-09	A	C	0.16	0.003	0.013	7.98E-01
13	rs9592461	A	G	0.49	0.024	0.002	1.17E-28	A	G	0.52	0.010	0.009	2.91E-01
10	rs997934	T	C	0.38	0.016	0.002	4.60E-13	T	C	0.39	-0.013	0.009	1.80E-01

Chr indicates chromosome; SNP, single nucleotide polymorphism; EA, effect allele; NEA, non-effect allele; EAF, effect allele frequency; SE, standard error.

[a] Beta presents natural logarithm of odds ratio.

[*] Palindromic SNPs with minor allele frequency of >0.42 were considered to be strand-ambiguous and removed from final analysis.

ESM Table 4. Characteristics of the single-nucleotide polymorphisms associated with major depressive disorder and their associations with heart failure

		Major depressive disorder						Heart failure							
Chr	SNP	EA	NEA	EAF	Beta[a]	SE	P	EA	NEA	EAF	Beta[a]	SE	P	Traits associated with outlier SNP[#]	Ambiguous palindrome[*]
1	rs1002656	T	C	0.70	-0.02	0.0024	4.84E-19	T	C	0.7148	-0.003	0.0088	0.73		
5	rs10061069	C	G	0.22	-0.03	0.0026	5.17E-22	C	G	0.2076	0.010	0.0097	0.32		
14	rs10149470	A	G	0.49	-0.02	0.0021	6.88E-22	A	G	0.4926	-0.005	0.0079	0.50		
10	rs1021363	A	G	0.35	0.02	0.0023	3.77E-23	A	G	0.3485	0.015	0.0082	0.07		
14	rs1045430	T	G	0.48	-0.02	0.0022	4.44E-25	T	G	0.4776	0.002	0.0079	0.85		
1	rs10789214	T	C	0.57	0.01	0.0022	3.81E-10	T	C	0.5643	0.022	0.0080	0.01		
9	rs10817969	T	G	0.72	0.02	0.0024	1.32E-12	T	G	0.7184	0.014	0.0087	0.11		
1	rs10890020	A	G	0.52	-0.03	0.0022	3.31E-34	A	G	0.508	-0.008	0.0079	0.35		
1	rs10913112	T	C	0.38	-0.02	0.0023	1.72E-20	T	C	0.3855	-6.00E-04	0.0083	0.95		
3	rs1095626	T	C	0.58	-0.03	0.0022	6.31E-39	T	C	0.5833	0.014	0.0079	0.08		
5	rs11135349	A	C	0.47	-0.03	0.0022	2.18E-30	A	C	0.4539	0.006	0.0079	0.42		
20	rs112768123	G	C	0.18	0.02	0.0028	7.23E-18	G	C	0.1984	-0.002	0.0098	0.83		
1	rs113188507	A	G	0.28	0.02	0.0025	2.94E-21	A	G	0.2786	0.009	0.0087	0.29		
14	rs1152578	T	C	0.44	-0.02	0.0022	4.19E-12	T	C	0.4395	0.012	0.0079	0.14		
1	rs11579246	A	G	0.91	0.03	0.0037	8.07E-18	A	G	0.8973	0.010	0.0133	0.44		
2	rs12052908	A	T	0.53	-0.02	0.0022	3.98E-22	A	T	0.5242	-0.019	0.0079	0.02		Yes
2	rs1226412	T	C	0.79	0.02	0.0027	3.35E-18	T	C	0.7982	-0.001	0.0098	0.92		

20	rs12624433	A	G	0.26	0.02	0.0025	2.16E-14	A	G	0.2533	0.019	0.0090	0.04		
16	rs12923444	A	C	0.56	-0.02	0.0023	1.76E-24	A	C	0.5628	0.005	0.0079	0.53		
18	rs12966052	C	G	0.18	-0.02	0.0028	3.76E-11	C	G	0.1818	0.007	0.0101	0.51		
18	rs12967143	C	G	0.70	-0.03	0.0024	1.61E-27	C	G	0.7148	-0.020	0.0087	0.02	Unipolar depression<br>Wellbeing measurement	
18	rs12967855	A	G	0.33	0.02	0.0023	1.32E-19	A	G	0.329	0.003	0.0083	0.73		
3	rs13058913	T	A	0.70	-0.02	0.0024	4.87E-12	T	A	0.6819	0.009	0.0084	0.30		
3	rs13084037	A	G	0.77	-0.02	0.0025	1.79E-17	A	G	0.7765	-0.011	0.0094	0.26		
13	rs1343605	A	C	0.38	0.02	0.0022	1.62E-25	A	C	0.3802	0.012	0.0081	0.13		
9	rs1354115	A	C	0.62	0.02	0.0022	4.39E-17	A	C	0.6341	-0.003	0.0081	0.70		
13	rs1409379	T	C	0.76	0.02	0.0025	8.26E-11	T	C	0.7615	0.003	0.0092	0.76		
11	rs1448938	A	G	0.42	0.02	0.0022	3.74E-15	A	G	0.4134	0.015	0.0079	0.05		
1	rs1466887	T	C	0.55	-0.01	0.0022	1.37E-08	T	C	0.5524	0.007	0.0079	0.39		
3	rs1554599	G	A	0.39	0.02	0.0022	1.18E-15	G	A	0.4225	-0.007	0.0079	0.40		
2	rs1568452	T	C	0.39	0.03	0.0022	3.08E-40	T	C	0.3714	-0.002	0.0081	0.77		
7	rs16887442	T	C	0.43	0.01	0.0022	1.25E-11	T	C	0.4316	0.009	0.0079	0.28		
1	rs169235	A	G	0.75	-0.02	0.0025	1.61E-11	A	G	0.751	-9.00E-04	0.0090	0.92		
1	rs17641524	T	C	0.21	-0.02	0.0027	7.90E-20	T	C	0.2292	0.008	0.0095	0.39		
1	rs1890946	T	C	0.47	-0.02	0.0022	8.62E-19	T	C	0.474	-0.009	0.0078	0.23		
6	rs1933802	C	G	0.45	-0.02	0.0022	1.36E-19	C	G	0.4585	0.004	0.0078	0.63		Yes
14	rs1956373	T	G	0.74	-0.02	0.0025	6.08E-12	T	G	0.7513	-0.005	0.0091	0.57		
11	rs198457	T	C	0.19	-0.02	0.0028	5.41E-14	T	C	0.1755	0.005	0.0106	0.66		
6	rs200949	A	G	0.87	0.04	0.0034	2.13E-37	A	G	0.875	-0.010	0.0151	0.49		
6	rs2029865	A	T	0.45	-0.02	0.0022	4.02E-17	A	T	0.4587	-0.011	0.0078	0.15		Yes
7	rs2043539	A	G	0.42	0.02	0.0022	8.32E-24	A	G	0.4116	0.021	0.0079	0.01	Unipolar depression	

7	rs2247523	C	G	0.53	-0.02	0.0021	9.05E-14	C	G	0.5314	0.011	0.0078	0.16		Yes
11	rs2509805	T	C	0.32	0.02	0.0024	4.92E-16	T	C	0.3319	-0.004	0.0084	0.67		
1	rs2568958	A	G	0.62	0.03	0.0022	4.45E-52	A	G	0.6068	0.004	0.0080	0.60		
9	rs263645	A	T	0.54	0.02	0.0022	6.92E-16	A	T	0.5343	-0.003	0.0077	0.72		Yes
9	rs2670139	T	C	0.76	-0.02	0.0025	2.19E-12	T	C	0.7482	-0.011	0.0091	0.24		
6	rs2876520	C	G	0.53	-0.02	0.0022	2.65E-17	C	G	0.5268	-0.011	0.0079	0.17		Yes
1	rs301799	T	C	0.57	-0.03	0.0022	4.05E-31	T	C	0.5824	0.019	0.0079	0.02		
5	rs30266	A	G	0.33	0.03	0.0023	1.99E-45	A	G	0.3167	0.005	0.0084	0.52		
5	rs3099439	T	C	0.53	-0.02	0.0022	1.61E-21	T	C	0.5374	0.004	0.0079	0.62		
12	rs3213572	A	G	0.47	0.02	0.0021	5.51E-20	A	G	0.4609	0.017	0.0079	0.04	Unipolar depression	
15	rs34488670	T	C	0.79	-0.02	0.0027	4.69E-13	T	C	0.7865	0.005	0.0096	0.64		
9	rs34653192	C	G	0.32	-0.02	0.0024	3.39E-16	C	G	0.333	-0.007	0.0086	0.39		
4	rs34937911	T	C	0.88	0.02	0.0034	5.39E-13	T	C	0.8791	0.008	0.0121	0.52		
4	rs35553410	T	C	0.75	-0.02	0.0025	2.18E-10	T	C	0.7444	0.005	0.0090	0.55		
9	rs3793577	A	G	0.47	-0.02	0.0022	5.58E-22	A	G	0.4701	0.009	0.0080	0.26		
7	rs3823624	T	C	0.81	0.03	0.0028	1.61E-26	T	C	0.8065	0.022	0.0102	0.03	Sleep duration<br>Unipolar depression	
4	rs45510091	A	G	0.95	0.05	0.0049	7.74E-21	A	G	0.9486	0.033	0.0182	0.07		
13	rs4772087	T	C	0.37	0.02	0.0023	2.34E-20	T	C	0.3907	-0.005	0.0081	0.53		
12	rs56314503	T	G	0.75	-0.02	0.0025	1.28E-16	T	G	0.7671	0.014	0.0094	0.15		
16	rs56887639	A	G	0.73	-0.02	0.0024	6.04E-13	A	G	0.7346	-0.010	0.0088	0.27		
11	rs57344483	A	G	0.93	-0.02	0.0041	5.26E-09	A	G	0.9214	0.025	0.0150	0.10		
7	rs58104186	A	G	0.47	0.02	0.0022	3.56E-18	A	G	0.4594	0.011	0.0079	0.18		
11	rs58621819	A	T	0.79	-0.02	0.0027	2.17E-10	A	T	0.7958	0.004	0.0115	0.73		
22	rs5995992	T	C	0.72	-0.03	0.0024	2.60E-35	T	C	0.7167	0.001	0.0088	0.88		
5	rs60157091	T	C	0.52	0.02	0.0022	5.54E-21	T	C	0.5121	-0.005	0.0078	0.53		
11	rs61902811	A	G	0.37	-0.03	0.0022	5.17E-39	A	G	0.3565	-0.010	0.0082	0.22		

14	rs61990288	A	G	0.51	-0.02	0.0021	1.96E-31	A	G	0.4944	-0.015	0.0078	0.05	Unipolar depression
18	rs62091461	T	C	0.23	-0.02	0.0026	1.75E-14	T	C	0.2461	-0.006	0.0092	0.55	
2	rs62188629	A	G	0.31	0.02	0.0024	6.15E-13	A	G	0.3208	-0.001	0.0086	0.89	
8	rs67436663	C	G	0.24	-0.01	0.0025	2.83E-08	C	G	0.2335	0.002	0.0139	0.87	
3	rs6783233	T	C	0.28	0.02	0.0024	1.14E-13	T	C	0.2807	0.010	0.0087	0.25	
9	rs7030813	T	C	0.37	0.03	0.0022	8.88E-30	T	C	0.3901	-0.007	0.0081	0.39	
11	rs7117514	A	G	0.54	-0.02	0.0022	3.64E-14	A	G	0.5364	0.011	0.0080	0.18	
16	rs7193263	A	G	0.67	-0.02	0.0023	3.83E-19	A	G	0.6653	-0.001	0.0084	0.90	
16	rs7198928	T	C	0.62	0.02	0.0022	3.71E-23	T	C	0.6172	-0.017	0.0094	0.06	
16	rs7200826	T	C	0.26	0.03	0.0025	3.09E-28	T	C	0.2716	0.016	0.0088	0.08	Unipolar depressionWellbeing measurementNeuroticism measurementDepressive symptom measurement
18	rs7227069	A	G	0.43	0.02	0.0022	2.13E-28	A	G	0.448	0.002	0.0078	0.84	
18	rs7241572	A	G	0.20	0.02	0.0027	2.56E-13	A	G	0.2046	-0.009	0.0099	0.34	
6	rs725616	T	C	0.36	0.01	0.0022	1.06E-09	T	C	0.361	0.007	0.0081	0.42	
1	rs72710803	A	C	0.91	-0.02	0.0038	1.35E-10	A	C	0.9195	0.004	0.0147	0.81	
17	rs75581564	A	G	0.12	0.02	0.0034	3.49E-13	A	G	0.1114	-0.011	0.0126	0.40	
2	rs7585722	T	C	0.85	-0.02	0.003	5.47E-13	T	C	0.8489	0.015	0.0110	0.17	
4	rs7685686	A	G	0.58	0.02	0.0022	5.52E-15	A	G	0.5703	-0.001	0.0080	0.90	
6	rs7758630	A	T	0.41	-0.02	0.0022	7.79E-14	A	T	0.3885	-0.015	0.0081	0.06	
7	rs7807677	T	C	0.55	0.02	0.0022	5.40E-22	T	C	0.5754	0.004	0.0080	0.65	
12	rs78337797	T	G	0.88	0.02	0.0034	1.37E-11	T	G	0.8788	0.002	0.0123	0.90	
8	rs7837935	T	G	0.15	-0.02	0.0031	1.15E-13	T	G	0.1462	-0.009	0.0111	0.43	

11	rs7932640	T	C	0.44	0.02	0.0022	3.40E-25	T	C	0.46	-0.002	0.0080	0.83
15	rs8037355	T	C	0.56	-0.02	0.0022	1.52E-18	T	C	0.5454	-0.005	0.0078	0.52
9	rs913930	A	G	0.64	-0.02	0.0023	1.03E-21	A	G	0.6335	-0.008	0.0083	0.32
6	rs9342504	T	C	0.60	0.02	0.0023	2.50E-12	T	C	0.6013	-0.001	0.0080	0.88
13	rs9545360	A	C	0.18	-0.02	0.0029	6.70E-09	A	C	0.1798	-8.00E-04	0.0118	0.95
9	rs958538	T	C	0.76	0.02	0.0026	1.28E-15	T	C	0.7584	3.00E-04	0.0091	0.97
13	rs9592461	A	G	0.49	0.02	0.0022	1.17E-28	A	G	0.4847	0.006	0.0079	0.49
10	rs997934	T	C	0.38	0.02	0.0023	4.60E-13	T	C	0.3836	0.008	0.0081	0.31

Chr indicates chromosome; SNP, single nucleotide polymorphism; EA, effect allele; NEA, non-effect allele; EAF, effect allele frequency; SE, standard error.

[a] Beta presents natural logarithm of odds ratio.

* Palindromic SNPs with minor allele frequency of >0.42 were considered to be strand-ambiguous and removed from final analysis.

# Six outlier SNPs were detected in leave-one-out analysis. Traits associated with thsoe six SNPs were searched in GWAS catalog and presented here.

ESM Table 5. Characteristics of the single-nucleotide polymorphisms associated with type 2 diabetes and their associations with major depressive disorder

		Type 2 diabetes						Major depressive disorder								
Chr	SNP	EA	NEA	EAF	Beta[a]	SE	P	EA	NEA	EAF	Beta[a]	SE	P	Proxy[#]	R^2	Ambiguous Palindrome*
15	rs1005752	A	C	0.72	0.03	0.004	1.40E-21	A	C	0.72	-0.002	0.005	6.48E-01			
8	rs10096633	C	T	0.88	0.03	0.004	2.90E-09	C	T	0.88	0.003	0.007	6.59E-01			
8	rs10097617	T	C	0.48	0.02	0.004	5.60E-12	T	C	0.48	-0.005	0.004	2.11E-01			
2	rs10193538	T	G	0.61	0.02	0.002	2.50E-09	T	G	0.61	-0.019	0.004	1.54E-05			
2	rs10195252	T	C	0.59	0.03	0.002	7.40E-18	T	C	0.59	-0.004	0.004	4.28E-01			
7	rs10228066	T	C	0.54	0.03	0.004	2.90E-21	T	C	0.55	-0.007	0.004	1.07E-01			
19	rs10406327	C	G	0.52	0.02	0.004	3.30E-09	C	G	0.52	0.000	0.004	9.88E-01			Yes
19	rs10406431	A	G	0.56	0.03	0.004	8.20E-17	A	G	0.56	0.008	0.004	7.81E-02			
12	rs1042725	T	C	0.49	0.03	0.002	8.00E-15	T	C	0.49	0.013	0.004	3.06E-03			
17	rs1043246	G	C	0.16	0.03	0.004	7.90E-12	G	C	0.16	-0.005	0.006	4.68E-01			
11	rs1061810	A	C	0.29	0.02	0.002	3.90E-09	A	C	0.29	-0.005	0.005	3.36E-01			
11	rs10750397	A	G	0.28	0.02	0.002	1.90E-08	A	G	0.28	0.004	0.005	4.29E-01			
9	rs10811660	G	A	0.83	0.07	0.004	2.20E-61	G	A	0.83	-0.002	0.006	7.15E-01			
11	rs10830963	G	C	0.28	0.05	0.004	1.70E-40	G	C	0.27	0.010	0.005	4.96E-02			
12	rs10842994	C	T	0.81	0.03	0.004	2.50E-17	C	T	0.80	0.009	0.005	1.06E-01			
10	rs10882101	T	C	0.59	0.05	0.002	5.60E-50	T	C	0.59	-0.004	0.004	4.08E-01			
11	rs10893829	T	C	0.85	0.03	0.004	5.10E-09	T	C	0.86	-0.003	0.006	6.33E-01			
17	rs10908278	T	A	0.48	0.03	0.004	1.30E-25	T	A	0.48	-0.002	0.004	6.13E-01			Yes
4	rs10937721	C	G	0.59	0.04	0.004	1.30E-33	C	G	0.59	0.009	0.005	5.62E-02			
17	rs10962	C	G	0.23	0.02	0.004	4.40E-08	C	G	0.22	0.003	0.005	5.59E-01			
9	rs10974438	C	A	0.36	0.03	0.002	1.70E-13	C	A	0.35	0.000	0.005	9.68E-01			
15	rs11070332	A	G	0.36	0.02	0.004	9.40E-12	A	G	0.35	0.009	0.005	4.67E-02			
10	rs1112718	A	G	0.60	0.05	0.004	2.70E-47	A	G	0.60	-0.001	0.004	7.72E-01			
10	rs11202627	T	C	0.15	0.03	0.004	2.00E-08	T	C	0.16	-0.005	0.006	3.91E-01			
6	rs112498319	C	A	0.41	0.02	0.002	3.90E-10	C	A	0.41	0.007	0.004	1.31E-01			

10	rs11257655	T	C	0.22	0.04	0.004	3.80E-24	T	C	0.21	0.002	0.006	6.94E-01	
1	rs1127215	C	T	0.58	0.02	0.002	5.10E-10	C	T	0.58	0.002	0.004	6.18E-01	
5	rs115505614	T	C	0.05	0.08	0.009	1.10E-24	T	C	0.05	-0.017	0.010	8.85E-02	
2	rs11688931	C	G	0.85	0.03	0.004	2.20E-09	C	G	0.85	0.000	0.006	9.83E-01	
20	rs11699802	C	T	0.54	0.02	0.004	1.70E-12	C	T	0.53	0.000	0.004	9.20E-01	
22	rs117001013	C	T	0.91	0.03	0.006	4.80E-08	C	T	0.92	0.007	0.008	3.67E-01	
3	rs11708067	A	G	0.77	0.04	0.004	9.30E-28	A	G	0.76	-0.002	0.005	6.93E-01	
3	rs11709077	G	A	0.88	0.04	0.004	9.00E-18	G	A	0.88	-0.006	0.007	3.45E-01	
6	rs11759026	G	A	0.23	0.03	0.004	6.10E-14	G	A	0.23	-0.006	0.005	2.78E-01	
11	rs11820019	T	C	0.97	0.06	0.009	9.10E-09	T	C	0.97	-0.008	0.014	5.38E-01	
3	rs11926707	C	T	0.63	0.02	0.004	4.40E-08	C	T	0.63	0.000	0.005	9.61E-01	
6	rs11967262	G	C	0.49	0.02	0.004	3.50E-09	G	C	0.49	0.003	0.004	4.66E-01	Yes
9	rs12001437	C	T	0.37	0.02	0.004	2.50E-08	C	T	0.37	-0.009	0.005	4.24E-02	
1	rs12140153	G	T	0.91	0.03	0.004	1.20E-08	G	T	0.91	0.000	0.008	9.74E-01	
18	rs12454712	T	C	0.61	0.03	0.002	4.70E-13	T	C	0.62	-0.004	0.005	4.45E-01	
2	rs1260326	C	T	0.61	0.03	0.002	9.60E-25	C	T	0.60	-0.004	0.004	3.69E-01	
12	rs12811407	A	G	0.33	0.02	0.004	2.40E-10	A	G	0.32	-0.004	0.005	3.74E-01	
15	rs12910825	G	A	0.36	0.03	0.004	8.10E-16	G	A	0.36	0.008	0.005	7.37E-02	
3	rs13085136	C	T	0.93	0.04	0.006	2.50E-09	C	T	0.93	-0.002	0.009	8.62E-01	
5	rs1316776	C	A	0.65	0.02	0.002	3.80E-09	C	A	0.65	0.010	0.005	2.43E-02	
8	rs13262861	C	A	0.83	0.05	0.004	3.20E-27	C	A	0.82	0.003	0.006	5.84E-01	
13	rs1359790	G	A	0.72	0.04	0.002	3.80E-25	G	A	0.71	0.001	0.005	9.10E-01	
15	rs13737	G	T	0.76	0.02	0.004	5.20E-10	G	T	0.75	0.007	0.005	1.89E-01	
17	rs1377807	C	G	0.31	0.03	0.004	1.70E-13	C	G	0.31	-0.007	0.005	1.22E-01	
9	rs1412234	C	T	0.32	0.02	0.002	3.40E-09	C	T	0.32	-0.005	0.005	2.98E-01	
9	rs1412830	C	T	0.63	0.02	0.004	8.80E-11	C	T	0.63	0.002	0.005	7.19E-01	
16	rs1421085	C	T	0.42	0.05	0.004	1.10E-60	C	T	0.41	0.010	0.004	2.01E-02	
12	rs1426371	G	A	0.74	0.03	0.002	3.30E-11	G	A	0.74	-0.004	0.005	4.71E-01	
11	rs145678014	G	T	0.96	0.05	0.008	1.60E-08	G	T	0.96	0.045	0.011	2.34E-05	
5	rs146886108	C	T	0.99	0.17	0.023	6.70E-14	C	T	0.99	0.017	0.028	5.59E-01	

8	rs148766658	C	T	0.04	0.05	0.010	3.20E-10	C	T	0.04	0.017	0.012	1.42E-01	
8	rs149364428	A	G	0.01	0.12	0.018	1.20E-11	A	G	0.01	-0.017	0.025	4.98E-01	
1	rs1493694	T	C	0.11	0.04	0.006	8.00E-14	T	C	0.11	0.007	0.007	3.41E-01	
4	rs1531583	T	G	0.05	0.05	0.008	1.10E-10	T	G	0.04	-0.002	0.011	8.81E-01	
7	rs1562396	G	A	0.32	0.03	0.004	8.10E-14	G	A	0.32	0.002	0.005	7.34E-01	
4	rs1580278	C	A	0.47	0.02	0.004	1.90E-08	C	A	0.47	0.000	0.004	9.29E-01	
3	rs17013314	G	A	0.03	0.05	0.010	1.00E-08	G	A	0.03	-0.011	0.013	4.09E-01	
7	rs1708302	C	T	0.51	0.04	0.002	6.40E-31	C	T	0.50	0.003	0.004	5.07E-01	
7	rs17168486	T	C	0.18	0.03	0.004	3.10E-14	T	C	0.18	0.002	0.006	7.51E-01	
5	rs17250977	G	A	0.04	0.06	0.009	1.20E-12	G	A	0.04	-0.011	0.012	3.29E-01	
8	rs17689007	G	A	0.53	0.02	0.004	2.50E-11	G	A	0.53	-0.006	0.005	1.74E-01	
10	rs177045	G	A	0.32	0.02	0.004	2.20E-10	G	A	0.32	0.000	0.005	9.26E-01	
9	rs17791513	A	G	0.93	0.05	0.006	3.00E-12	A	G	0.94	0.006	0.009	5.19E-01	
3	rs17819328	G	T	0.42	0.02	0.004	2.60E-08	G	T	0.42	0.001	0.004	8.10E-01	
11	rs1783541	T	C	0.20	0.03	0.004	5.20E-12	T	C	0.21	0.027	0.005	3.37E-07	
14	rs17836088	C	G	0.22	0.03	0.002	6.90E-13	C	G	0.22	0.005	0.005	3.57E-01	
12	rs1796330	G	C	0.57	0.02	0.004	2.50E-11	G	C	0.56	-0.006	0.004	1.41E-01	Yes
12	rs1800574	T	C	0.03	0.06	0.009	3.50E-11	T	C	0.03	-0.028	0.013	3.28E-02	
20	rs1800961	T	C	0.04	0.07	0.009	1.10E-16	T	C	0.03	0.004	0.012	7.56E-01	
4	rs1801212	A	G	0.71	0.04	0.004	1.30E-34	A	G	0.72	0.006	0.005	2.00E-01	
22	rs1801645	C	T	0.28	0.03	0.002	1.20E-10	C	T	0.26	0.011	0.005	3.01E-02	
10	rs180988137	G	A	0.01	0.14	0.017	2.40E-16	G	A	0.01	-0.012	0.023	5.92E-01	
10	rs184509201	C	G	0.98	0.08	0.013	3.10E-11	C	G	0.98	-0.001	0.017	9.73E-01	
2	rs2028150	C	G	0.60	0.02	0.004	5.10E-11	C	G	0.59	0.007	0.004	9.05E-02	
12	rs2197973	T	C	0.54	0.02	0.004	9.50E-09	T	C	0.54	0.003	0.004	4.44E-01	
11	rs2237895	C	A	0.43	0.05	0.002	3.60E-42	C	A	0.42	0.002	0.005	7.34E-01	
19	rs2238689	C	T	0.42	0.03	0.002	8.70E-15	C	T	0.41	0.002	0.004	6.36E-01	
2	rs2249105	A	G	0.63	0.02	0.004	1.40E-11	A	G	0.63	0.002	0.005	7.14E-01	
12	rs2258238	T	A	0.10	0.05	0.004	3.30E-23	T	A	0.11	0.000	0.007	9.74E-01	
10	rs2280141	T	G	0.52	0.02	0.004	2.70E-08	T	G	0.53	0.000	0.004	9.69E-01	

11	rs2283220	A	G	0.69	0.02	0.004	1.40E-09	A	G	0.69	-0.001	0.005	9.13E-01
5	rs2307111	T	C	0.61	0.02	0.004	6.50E-13	T	C	0.61	0.002	0.004	6.33E-01
11	rs231361	A	G	0.26	0.03	0.004	2.40E-15	A	G	0.26	0.000	0.005	9.96E-01
2	rs243024	A	G	0.46	0.03	0.002	7.20E-15	A	G	0.45	0.005	0.004	2.59E-01
15	rs2456530	T	C	0.13	0.03	0.004	1.30E-09	T	C	0.13	0.002	0.006	8.19E-01
10	rs2642588	G	T	0.70	0.02	0.004	2.30E-09	G	T	0.70	0.001	0.005	8.36E-01
9	rs2796441	G	A	0.59	0.03	0.004	4.60E-21	G	A	0.58	0.002	0.004	6.96E-01
6	rs2800733	A	G	0.72	0.03	0.002	4.30E-12	A	G	0.71	0.003	0.005	5.38E-01
1	rs2820446	C	G	0.71	0.02	0.004	2.70E-10	C	G	0.70	-0.003	0.005	5.48E-01
9	rs28505901	G	A	0.75	0.03	0.004	7.20E-19	G	A	0.76	-0.004	0.005	3.97E-01
1	rs291367	G	A	0.63	0.02	0.004	3.40E-08	G	A	0.63	-0.001	0.005	8.08E-01
16	rs2925979	T	C	0.30	0.02	0.004	3.90E-08	T	C	0.30	0.000	0.005	9.47E-01
2	rs2972144	G	A	0.64	0.04	0.004	3.70E-37	G	A	0.64	-0.001	0.005	9.05E-01
19	rs3111316	A	G	0.59	0.02	0.004	1.60E-12	A	G	0.59	-0.005	0.004	2.72E-01
12	rs3217792	C	T	0.91	0.05	0.006	9.40E-20	C	T	0.91	0.001	0.008	9.02E-01
12	rs3217860	G	A	0.26	0.03	0.002	6.10E-11	G	A	0.25	0.000	0.005	9.45E-01
5	rs329122	A	G	0.43	0.02	0.004	3.30E-08	A	G	0.42	-0.012	0.004	4.34E-03
1	rs340874	C	T	0.56	0.03	0.004	3.80E-21	C	T	0.56	-0.014	0.004	1.43E-03
15	rs34715063	C	T	0.12	0.04	0.004	6.30E-13	C	T	0.13	-0.005	0.007	4.32E-01
1	rs348330	G	A	0.36	0.02	0.004	8.40E-11	G	A	0.37	0.008	0.005	6.65E-02
17	rs34855406	C	G	0.28	0.02	0.004	1.00E-09	C	G	0.28	0.001	0.005	8.87E-01
3	rs35352848	T	C	0.79	0.04	0.004	2.90E-20	T	C	0.80	-0.005	0.005	3.41E-01
17	rs35895680	C	A	0.68	0.03	0.002	5.70E-12	C	A	0.67	-0.001	0.005	9.07E-01
2	rs35913461	C	T	0.83	0.03	0.004	3.70E-09	C	T	0.83	0.005	0.006	3.98E-01
1	rs3768321	T	G	0.20	0.04	0.004	3.30E-20	T	G	0.20	0.005	0.005	3.89E-01
3	rs3774723	G	A	0.84	0.03	0.004	1.10E-10	G	A	0.85	0.009	0.006	1.29E-01
6	rs3798519	C	A	0.18	0.03	0.004	4.70E-12	C	A	0.18	0.005	0.006	3.76E-01
8	rs3802177	G	A	0.69	0.05	0.004	3.10E-45	G	A	0.69	0.002	0.005	6.49E-01
19	rs3810291	A	G	0.67	0.03	0.002	5.80E-12	A	G	0.67	0.007	0.005	1.58E-01
3	rs3887925	T	C	0.55	0.03	0.002	2.10E-14	T	C	0.55	-0.013	0.004	3.38E-03

10	rs41277236	T	C	0.04	0.05	0.010	2.10E-09	T	C	0.04	0.009	0.011	4.31E-01	
12	rs4148856	C	G	0.78	0.02	0.004	1.40E-08	C	G	0.79	0.014	0.005	6.99E-03	
12	rs4238013	C	T	0.21	0.03	0.004	1.20E-10	C	T	0.20	0.004	0.005	4.45E-01	
7	rs4279506	G	C	0.61	0.02	0.004	1.50E-08	G	C	0.62	0.002	0.005	7.13E-01	
19	rs429358	T	C	0.85	0.03	0.004	2.40E-10	T	C	0.85	0.001	0.006	9.41E-01	
5	rs4457053	G	A	0.30	0.03	0.004	5.00E-14	G	A	0.32	0.001	0.005	8.19E-01	
5	rs465002	T	C	0.74	0.03	0.004	6.00E-20	T	C	0.75	-0.003	0.005	5.56E-01	
3	rs4686471	C	T	0.61	0.03	0.004	1.10E-19	C	T	0.61	0.000	0.004	9.60E-01	
3	rs4688760	T	C	0.68	0.02	0.002	3.70E-09	T	C	0.69	0.011	0.005	2.24E-02	
6	rs4709746	C	T	0.87	0.03	0.004	8.70E-09	C	T	0.87	0.001	0.006	8.71E-01	
8	rs4736819	T	C	0.55	0.03	0.002	1.10E-24	T	C	0.55	0.000	0.004	9.52E-01	
6	rs474513	A	G	0.52	0.02	0.004	8.10E-09	A	G	0.52	-0.004	0.004	3.30E-01	
19	rs4804833	A	G	0.39	0.03	0.002	3.30E-13	A	G	0.38	0.006	0.005	1.53E-01	
20	rs4810426	T	C	0.11	0.04	0.004	1.00E-14	T	C	0.10	0.000	0.007	9.71E-01	
17	rs4925109	A	G	0.32	0.02	0.004	3.50E-08	A	G	0.31	-0.008	0.005	7.88E-02	
11	rs4929965	A	G	0.38	0.03	0.002	2.00E-23	A	G	0.38	-0.002	0.005	6.17E-01	
15	rs4932265	T	C	0.27	0.03	0.004	6.60E-17	T	C	0.26	0.004	0.005	4.60E-01	
8	rs4977213	C	T	0.37	0.02	0.004	6.60E-12	C	T	0.38	-0.008	0.005	9.07E-02	
11	rs5213	C	T	0.36	0.03	0.002	6.80E-18	C	T	0.35	-0.006	0.005	2.20E-01	
18	rs523288	T	A	0.24	0.03	0.004	1.70E-12	T	A	0.24	-0.015	0.005	2.58E-03	
1	rs539515	C	A	0.20	0.03	0.004	2.60E-10	C	A	0.20	0.001	0.005	8.14E-01	
9	rs55653563	A	C	0.73	0.02	0.002	3.20E-08	A	C	0.73	-0.002	0.005	6.29E-01	
4	rs56337234	C	T	0.50	0.03	0.004	1.20E-15	C	T	0.51	-0.001	0.005	7.68E-01	
12	rs56348580	G	C	0.69	0.03	0.002	6.50E-13	G	C	0.69	0.003	0.005	5.59E-01	
11	rs57235767	C	T	0.71	0.02	0.004	2.90E-09	C	T	0.71	-0.004	0.005	4.21E-01	
1	rs58432198	C	T	0.88	0.03	0.004	3.90E-08	C	T	0.88	0.010	0.007	1.38E-01	
4	rs58730668	T	C	0.86	0.03	0.004	4.20E-10	T	C	0.86	0.008	0.006	2.28E-01	
6	rs601945	G	A	0.18	0.03	0.004	1.40E-15	G	A	0.19	-0.001	0.006	8.96E-01	
20	rs6063048	G	A	0.72	0.02	0.002	1.10E-08	G	A	0.72	0.002	0.005	6.45E-01	
20	rs6070625	G	C	0.52	0.02	0.004	5.10E-12	G	C	0.51	-0.005	0.004	2.56E-01	Yes

17	rs61676547	C	G	0.19	0.02	0.004	4.00E-08	C	G	0.19	0.011	0.006	4.62E-02			
2	rs62107261	T	C	0.95	0.05	0.010	3.60E-09	T	C	0.95	0.001	0.010	8.94E-01			
7	rs62492368	A	G	0.31	0.02	0.004	2.40E-10	A	G	0.31	0.004	0.005	4.54E-01			
6	rs6458354	C	T	0.29	0.02	0.004	3.40E-09	C	T	0.29	0.001	0.005	7.80E-01			
7	rs6459733	G	C	0.67	0.03	0.002	7.10E-17	G	C	0.66	0.001	0.005	8.61E-01			
22	rs6518681	G	A	0.91	0.04	0.006	1.70E-10	G	A	0.91	-0.004	0.008	6.16E-01			
16	rs6600191	T	C	0.82	0.03	0.004	1.60E-12	T	C	0.82	0.004	0.006	4.43E-01			
2	rs6708643	A	G	0.50	0.02	0.004	2.10E-08	A	G	0.51	0.004	0.004	4.15E-01			
11	rs67232546	T	C	0.21	0.03	0.004	3.00E-10	T	C	0.21	0.004	0.005	4.14E-01			
3	rs6780171	A	T	0.31	0.05	0.002	4.40E-45	A	T	0.31	0.002	0.005	7.34E-01			
5	rs6885132	C	G	0.90	0.04	0.006	1.60E-12	C	G	0.90	0.003	0.007	6.52E-01			
9	rs7022807	G	A	0.40	0.02	0.004	4.90E-09	G	A	0.40	0.000	0.004	9.93E-01			
5	rs702634	A	G	0.69	0.02	0.004	1.10E-09	A	G	0.69	-0.005	0.005	3.07E-01			
10	rs703972	G	C	0.53	0.03	0.002	1.90E-24	G	C	0.54	-0.006	0.004	2.08E-01			Yes
10	rs7078559	T	C	0.58	0.02	0.002	1.80E-10	T	C	0.59	0.007	0.004	9.25E-02			
11	rs7115753	A	G	0.45	0.02	0.004	2.60E-08	A	G	0.44	0.002	0.004	5.86E-01			
15	rs7178762	C	T	0.46	0.02	0.004	6.90E-09	C	T	0.45	0.008	0.004	6.63E-02			
12	rs718314	G	A	0.25	0.02	0.004	3.50E-10	G	A	0.24	0.002	0.005	6.32E-01			
17	rs7222481	C	G	0.32	0.02	0.002	1.50E-08	C	G	0.33	0.001	0.005	8.52E-01			
10	rs72631105	A	G	0.19	0.02	0.004	3.10E-08	A	G	0.19	0.004	0.006	4.30E-01			
16	rs72802342	C	A	0.92	0.06	0.008	5.10E-25	C	A	0.92	-0.005	0.008	5.30E-01			
12	rs73226260	G	A	0.97	0.05	0.010	1.60E-08	G	A	0.97	0.009	0.012	4.83E-01			
22	rs738408	T	C	0.23	0.02	0.004	1.70E-09	T	C	0.22	-0.001	0.005	8.84E-01			
3	rs74653713	C	A	0.96	0.05	0.010	1.50E-09	C	A	0.96	-0.007	0.011	5.47E-01			
9	rs76011118	A	G	0.03	0.09	0.009	6.80E-18	A	G	0.03	0.007	0.014	6.25E-01			
4	rs7669833	T	A	0.70	0.02	0.004	5.80E-11	T	A	0.71	0.000	0.005	9.97E-01			
12	rs76895963	T	G	0.98	0.20	0.014	4.80E-53	T	G	0.98	0.006	0.017	7.41E-01			
11	rs77464186	A	C	0.84	0.05	0.004	7.60E-27	A	C	0.84	-0.013	0.006	3.05E-02			
6	rs7756992	G	A	0.27	0.06	0.002	1.00E-64	G	A	0.27	0.000	0.005	9.51E-01			
10	rs78025551	C	G	0.85	0.07	0.006	9.90E-54	C	G	0.86	-0.016	0.006	1.07E-02			

10	rs7903146	T	C	0.30	0.14	0.005	1.00E-200	T	C	0.29	-0.012	0.005	1.17E-02			
1	rs79687284	C	G	0.03	0.08	0.009	4.30E-19	C	G	0.03	-0.011	0.012	3.68E-01			
2	rs80147536	A	T	0.90	0.06	0.006	3.80E-25	A	T	0.90	-0.012	0.007	1.02E-01			
15	rs8032939	C	T	0.25	0.02	0.002	3.60E-08	C	T	0.25	0.018	0.005	3.12E-04			
15	rs8037894	G	C	0.57	0.02	0.002	1.70E-09	G	C	0.57	-0.001	0.004	8.53E-01			Yes
19	rs8107974	T	A	0.08	0.05	0.006	8.70E-15	T	A	0.08	-0.017	0.008	4.12E-02			
16	rs862320	C	T	0.58	0.02	0.004	1.30E-12	C	T	0.59	0.010	0.004	1.70E-02			
7	rs878521	A	G	0.25	0.03	0.004	2.80E-11	A	G	0.25	0.006	0.005	2.38E-01			
6	rs9379084	G	A	0.89	0.05	0.004	5.60E-20	G	A	0.88	0.006	0.007	3.90E-01			
13	rs9563615	A	T	0.71	0.02	0.004	9.80E-10	A	T	0.71	-0.005	0.005	3.20E-01			
13	rs9569864	C	T	0.82	0.03	0.004	9.70E-10	C	T	0.83	-0.002	0.006	7.97E-01			
5	rs96844	G	A	0.26	0.02	0.002	2.80E-08	G	A	0.25	-0.001	0.005	8.17E-01			
5	rs9687832	A	G	0.20	0.03	0.004	2.20E-12	A	G	0.20	0.004	0.005	4.45E-01			
3	rs9860730	A	G	0.70	0.03	0.002	1.00E-11	A	G	0.70	0.001	0.005	8.58E-01			
3	rs9873618	G	A	0.71	0.03	0.002	1.20E-15	G	A	0.71	-0.009	0.005	5.64E-02			
18	rs72926932	C(T)	A(C)	0.08	0.04	0.004	2.30E-10	T	C	0.08	-0.013	0.008	1.10E-01	rs72926954	0.97	
2	rs11688682	G	C	0.73	0.03	0.004	5.40E-12	NA	NA	NA	NA	NA	NA		<0.90	
4	rs12642790	A	G	0.34	0.02	0.002	2.50E-09	NA	NA	NA	NA	NA	NA			
5	rs138337556	G	A	0.00	0.22	0.033	4.30E-11	NA	NA	NA	NA	NA	NA		<0.90	
5	rs78408340	G	C	0.01	0.17	0.019	5.30E-18	NA	NA	NA	NA	NA	NA		<0.90	
8	rs11786992	A	C	0.64	0.02	0.004	3.80E-08	NA	NA	NA	NA	NA	NA		<0.90	
10	rs536643418	G	C	0.01	0.22	0.040	3.80E-08	NA	NA	NA	NA	NA	NA			
11	rs571342427	C	T	0.00	0.27	0.048	1.70E-08	NA	NA	NA	NA	NA	NA			
11	rs2237897	C	T	0.95	0.09	0.007	1.30E-28	NA	NA	NA	NA	NA	NA			
11	rs67254669	G	A	0.00	0.29	0.051	3.20E-08	NA	NA	NA	NA	NA	NA		<0.90	
16	rs199795270	C	G	0.01	0.12	0.019	2.60E-08	NA	NA	NA	NA	NA	NA		<0.90	
2	rs562386202	G	A	0.00	0.51	0.092	3.40E-08	NA	NA	NA	NA	NA	NA			

Chr indicates chromosome; SNP, single nucleotide polymorphism; EA, effect allele; NEA, non-effect allele; EAF, effect allele frequency; SE, standard error.

[a] Beta presents natural logarithm of odds ratio.

[#] Genetic variants which were not available in the outcome summary statistics data were replaced by their proxies (R^2>0.90). The effect allele and non-effect allele of proxies were noted in the brackets.
[*] Palindromic SNPs with minor allele frequency of >0.42 were considered to be strand-ambiguous and removed from final analysis.

ESM Table 6. Characteristics of the single-nucleotide polymorphisms associated with coronary artery disease and their associations with major depressive disorder

		Coronary artery disease						Major depressive disorder						
Chr	SNP	EA	NEA	EAF	Beta[a]	SE	P	EA	NEA	EAF	Beta[a]	SE	P	Ambiguous palindrome[*]
1	rs11206510	T	C	0.85	0.08	0.014	2.34E-08	T	C	0.81	-0.012	0.006	2.45E-02	
1	rs6689306	A	G	0.45	0.06	0.010	2.60E-09	A	G	0.42	0.002	0.004	6.42E-01	
1	rs67180937	G	T	0.66	0.08	0.014	1.01E-12	G	T	0.74	-0.001	0.005	8.20E-01	
1	rs7528419	A	G	0.79	0.11	0.013	1.97E-23	A	G	0.78	0.000	0.005	9.31E-01	
1	rs9970807	C	T	0.92	0.12	0.018	5.00E-14	C	T	0.91	-0.002	0.008	7.99E-01	
2	rs16986953	A	G	0.10	0.09	0.014	1.45E-08	A	G	0.07	0.009	0.009	2.95E-01	
2	rs7568458	A	T	0.45	0.06	0.010	3.62E-10	A	T	0.45	0.000	0.004	9.56E-01	Yes
4	rs17087335	T	G	0.21	0.06	0.014	4.59E-08	T	G	0.19	0.008	0.006	1.60E-01	
4	rs4593108	C	G	0.80	0.07	0.014	8.82E-10	C	G	0.83	0.002	0.006	7.03E-01	
4	rs72689147	G	T	0.82	0.07	0.014	6.07E-09	G	T	0.82	0.008	0.006	1.48E-01	
6	rs12202017	A	G	0.70	0.07	0.009	1.98E-11	A	G	0.71	-0.006	0.005	2.22E-01	
6	rs4252185	C	T	0.06	0.29	0.026	1.64E-32	C	T	0.10	0.004	0.008	5.86E-01	
6	rs55730499	T	C	0.06	0.31	0.025	5.39E-39	T	C	0.08	-0.002	0.008	7.70E-01	
6	rs56336142	T	C	0.81	0.07	0.009	1.85E-08	T	C	0.79	-0.012	0.005	1.93E-02	
6	rs9349379	G	A	0.43	0.13	0.009	1.81E-42	G	A	0.41	-0.003	0.004	5.62E-01	
7	rs11556924	C	T	0.69	0.08	0.009	5.34E-11	C	T	0.62	0.006	0.004	1.49E-01	
7	rs2107595	A	G	0.20	0.08	0.009	8.05E-11	A	G	0.16	0.010	0.006	1.08E-01	
7	rs3918226	T	C	0.06	0.13	0.022	1.69E-09	T	C	0.08	0.000	0.008	9.63E-01	
9	rs2519093	T	C	0.19	0.08	0.014	1.19E-11	T	C	0.19	-0.007	0.006	2.12E-01	
9	rs2891168	G	A	0.49	0.19	0.012	2.29E-98	G	A	0.49	0.009	0.004	4.68E-02	
10	rs11191416	T	G	0.87	0.08	0.014	4.65E-09	T	G	0.91	0.032	0.008	3.07E-05	
10	rs1412444	T	C	0.37	0.07	0.009	5.15E-12	T	C	0.34	-0.001	0.005	7.55E-01	
10	rs1870634	G	T	0.64	0.08	0.009	5.55E-15	G	T	0.67	0.005	0.005	2.97E-01	
10	rs2487928	A	G	0.42	0.06	0.010	4.41E-11	A	G	0.45	0.004	0.004	3.92E-01	

11	rs10840293	A	G	0.55	0.06	0.010	1.28E-08	A	G	0.56	0.003	0.004	5.29E-01	
11	rs2128739	A	C	0.32	0.07	0.009	7.05E-11	A	C	0.28	0.001	0.005	7.92E-01	
12	rs2681472	G	A	0.20	0.08	0.009	6.17E-11	G	A	0.17	0.007	0.006	2.55E-01	
12	rs3184504	T	C	0.42	0.07	0.009	1.03E-09	T	C	0.48	-0.003	0.004	4.69E-01	
13	rs11838776	A	G	0.26	0.07	0.009	1.83E-10	A	G	0.28	-0.001	0.005	9.17E-01	
14	rs10139550	G	C	0.42	0.06	0.010	1.38E-08	G	C	0.42	0.000	0.004	9.33E-01	Yes
15	rs4468572	C	T	0.59	0.08	0.009	4.44E-16	C	T	0.57	0.006	0.004	2.08E-01	
15	rs56062135	C	T	0.79	0.07	0.014	4.52E-09	C	T	0.76	0.005	0.005	3.56E-01	
15	rs8042271	G	A	0.90	0.10	0.018	3.68E-08	G	A	0.96	0.001	0.011	9.16E-01	
17	rs7212798	C	T	0.15	0.08	0.014	1.88E-08	C	T	0.15	0.000	0.006	9.60E-01	
18	rs663129	A	G	0.26	0.06	0.010	3.20E-08	A	G	0.24	-0.016	0.005	1.66E-03	
19	rs4420638	G	A	0.17	0.10	0.014	7.07E-11	G	A	0.19	-0.001	0.006	8.72E-01	
19	rs56289821	G	A	0.90	0.13	0.018	4.44E-15	G	A	0.88	-0.004	0.007	5.48E-01	
21	rs28451064	A	G	0.12	0.13	0.013	1.33E-15	A	G	0.13	-0.002	0.007	8.00E-01	
22	rs180803	G	T	0.97	0.18	0.029	1.64E-10	G	T	0.99	0.016	0.021	4.44E-01	
2	rs192011340	D	I	0.75	0.07	0.014	2.89E-08	NA	NA	NA	NA	NA	NA	
2	chr2:44074126:D	I	D	0.74	0.06	0.014	2.60E-08	NA	NA	NA	NA	NA	NA	
2	rs17678683	G	T	0.09	0.10	0.018	3.00E-09	NA	NA	NA	NA	NA	NA	
2	rs201810558	I	D	0.11	0.14	0.013	2.15E-18	NA	NA	NA	NA	NA	NA	
3	rs377727514	I	D	0.16	0.08	0.009	2.89E-09	NA	NA	NA	NA	NA	NA	

Chr indicates chromosome; SNP, single nucleotide polymorphism; EA, effect allele; NEA, non-effect allele; EAF, effect allele frequency; SE, standard error.

a Beta presents natural logarithm of odds ratio.

* Palindromic SNPs with minor allele frequency of >0.42 were considered to be strand-ambiguous and removed from final analysis.

ESM Table 7. Characteristics of the single-nucleotide polymorphisms associated with heart failure and their associations with major depressive disorder

		Heart failure						Major depressive disorder						
Chr	SNP	EA	NEA	EAF	Beta[a]	SE	P	EA	NEA	EAF	Beta[a]	SE	P	Ambiguous palindrome*
5	rs11745324	A	G	0.23	-0.05	0.0095	2.35E-08	A	G	0.23	-0.006	0.0051	2.54E-01	
6	rs140570886	T	C	0.98	-0.21	0.0328	7.69E-11	T	C	0.98	0.006	0.018	7.31E-01	
9	rs1556516	C	G	0.48	0.06	0.0078	1.57E-15	C	G	0.50	0.009	0.0043	2.90E-02	Yes
4	rs17042102	A	G	0.12	0.11	0.0121	5.71E-20	A	G	0.11	0.001	0.007	9.23E-01	
10	rs17617337	T	C	0.22	-0.06	0.0095	3.65E-09	T	C	0.22	0.015	0.0052	3.84E-03	
6	rs4135240	T	C	0.66	0.05	0.0084	6.84E-09	T	C	0.66	0.006	0.0046	2.11E-01	
10	rs4746140	C	G	0.15	-0.07	0.0109	1.10E-09	C	G	0.15	0.000	0.006	9.52E-01	
12	rs4766578	A	T	0.53	-0.04	0.0079	4.90E-08	A	T	0.50	0.003	0.0043	4.88E-01	Yes
6	rs55730499	T	C	0.07	0.11	0.0157	1.83E-11	T	C	0.08	-0.002	0.0081	7.70E-01	
16	rs56094641	A	G	0.58	-0.05	0.008	1.21E-08	A	G	0.59	-0.010	0.0044	1.69E-02	
9	rs600038	T	C	0.79	-0.06	0.0096	3.68E-09	T	C	0.79	0.003	0.0053	6.04E-01	
1	rs660240	T	C	0.21	-0.06	0.0097	3.25E-10	T	C	0.21	-0.002	0.0052	7.68E-01	

Chr indicates chromosome; SNP, single nucleotide polymorphism; EA, effect allele; NEA, non-effect allele; EAF, effect allele frequency; SE, standard error.

a Beta presents natural logarithm of odds ratio.

* Palindromic SNPs with minor allele frequency of >0.42 were considered to be strand-ambiguous and removed from final analysis.

ESM Table 8. Power calculation for the association estimates in Mendelian Randomization analyses

Exposure	Estimated r-square for exposure*	Outcome	Participants in outcome GWAS data (proportion of cases, %)	Power to detect OR of following magnitude			
				1.10	1.20	1.30	1.40
Major depressive disorder	0.003	Type 2 diabetes	898130 (8.25)	0.29	0.80	0.99	1.00
		Coronary artery disease	184305 (32.99)	0.19	0.55	0.86	0.98
		Heart failure	977323 (4.84)	0.21	0.63	0.93	1.00
Type 2 diabetes	0.01	Major depressive disorder	500199 (34.14)	0.90	1.00	1.00	1.00
Coronary artery disease	0.017			0.99	1.00	1.00	1.00
Heart failure	0.0024			0.36	0.87	0.99	1.00

GWAS indicates genome-wide association study; OR, odds ratio.

* The variance of the exposure trait explained by genetic variants (r-square) was computed using get-or-from-lor commend in TwoSampleMR package [https://github.com/MRCIEU/TwoSampleMR] in R 3.6.0, based on the parameters of effect size estimates (log(OR)), effect allele frequency, sample size of cases and controls, and prevalence of exposure trait. As prevalence contributed little to the calculation, we assumed all the prevalence of traits to be 10%.

ESM Fig. 1. Scatter plot and leave-one-out analysis for the association of major depressive disorder with type 2 diabetes

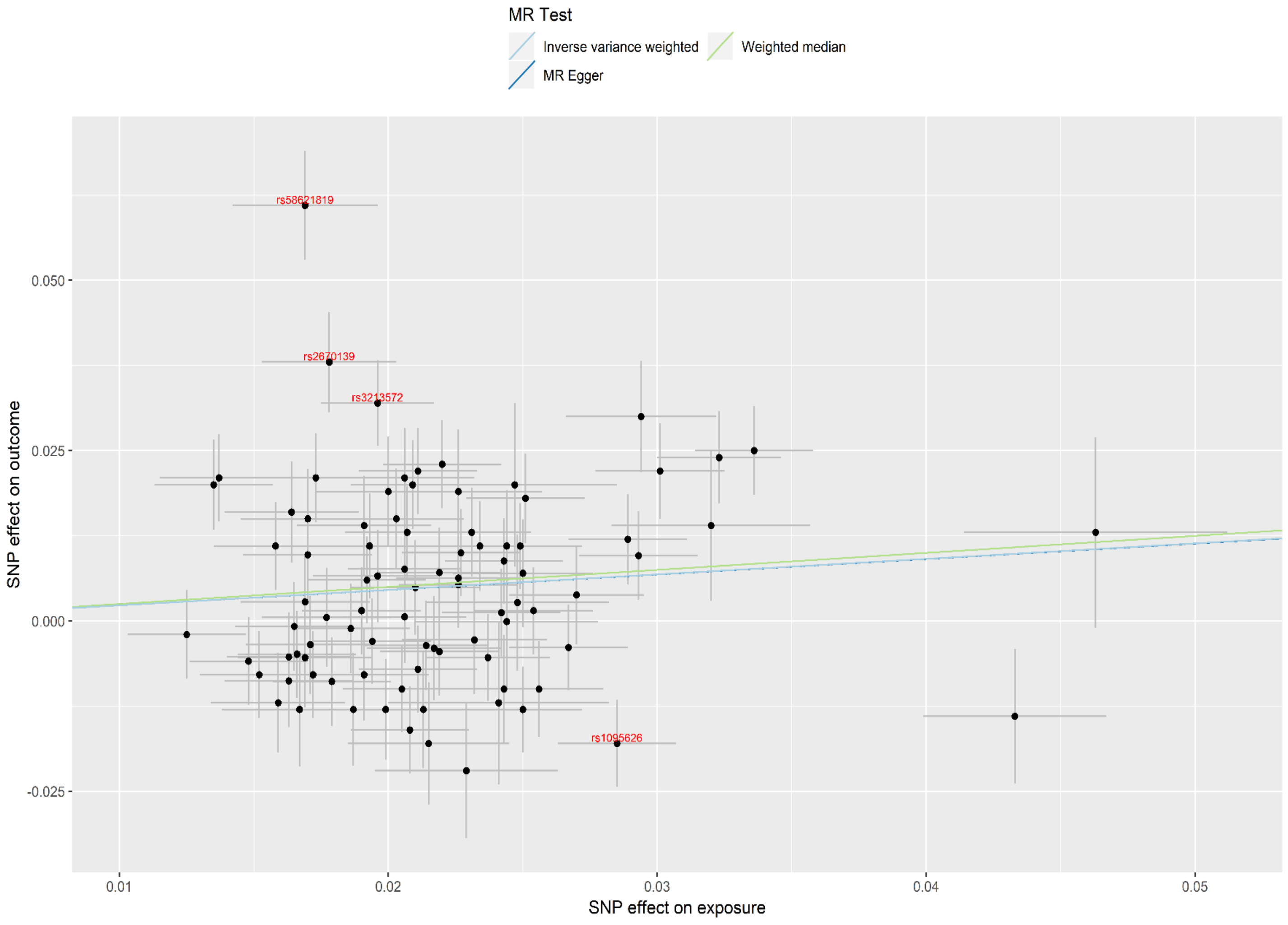

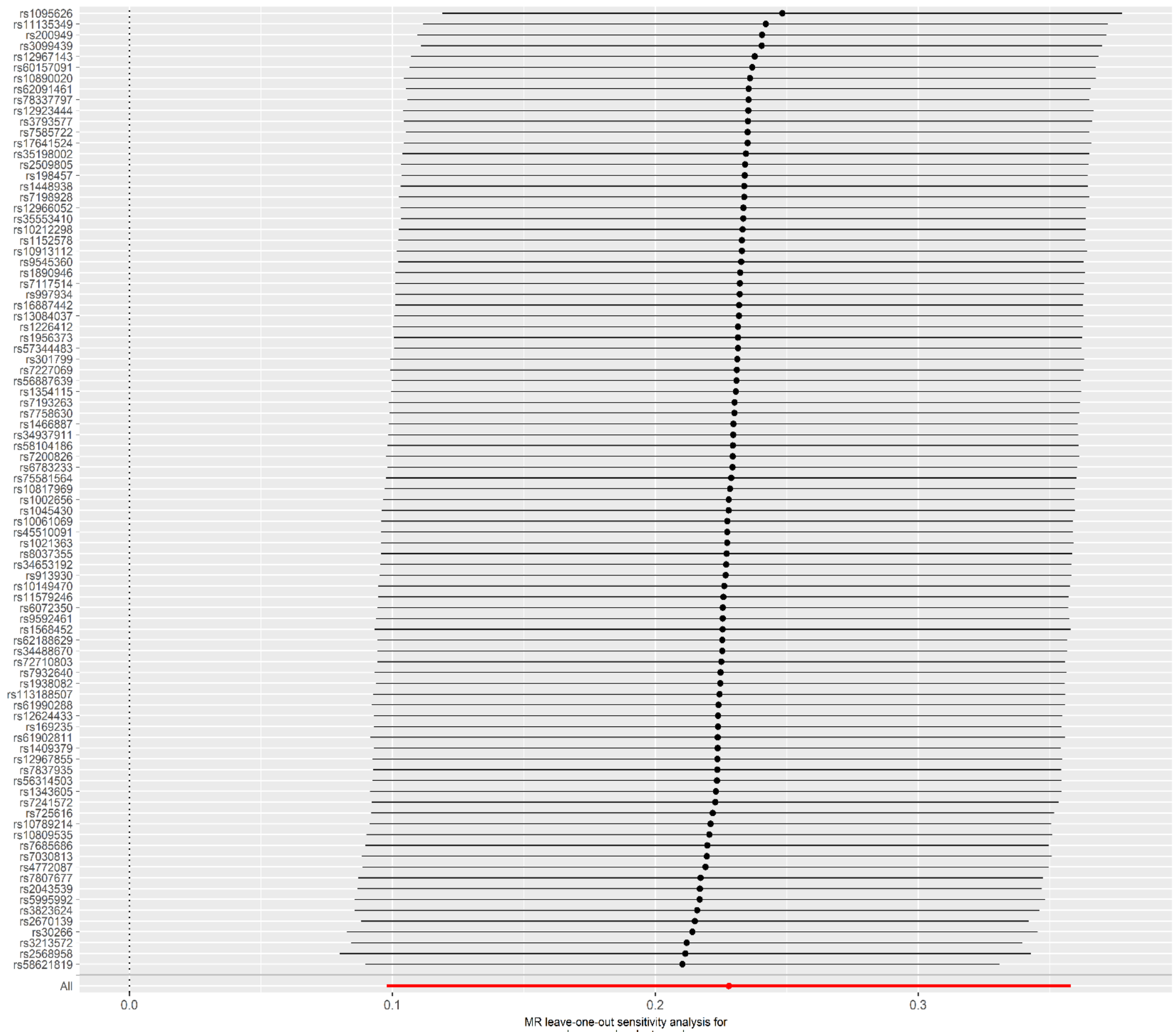

SNP indicates single nucleotide polymorphism; MR, mendelian randomization.

In the scatter plot, the red marked SNPs were those identified as possible outliers in the MR-PRESSO analysis.

ESM Fig. 2. The association of major depressive disorder with type 2 diabetes using type 2 diabetes summary statistics data with adjustment for BMI

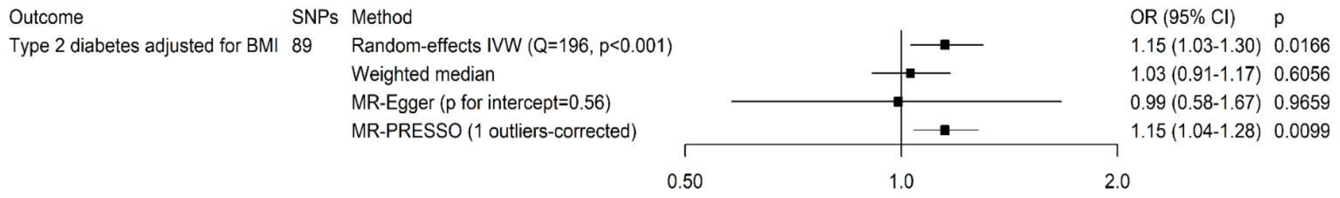

CI indicates confidence interval; IVW, inverse-variance weighted; OR, odds ratio; BMI, body mass index; MR-PRESSO, Mendelian randomization pleiotropy residual sum and outlier.

ESM Fig. 3. Scatter plot and leave-one-out analysis for the association of major depressive disorder with coronary artery disease

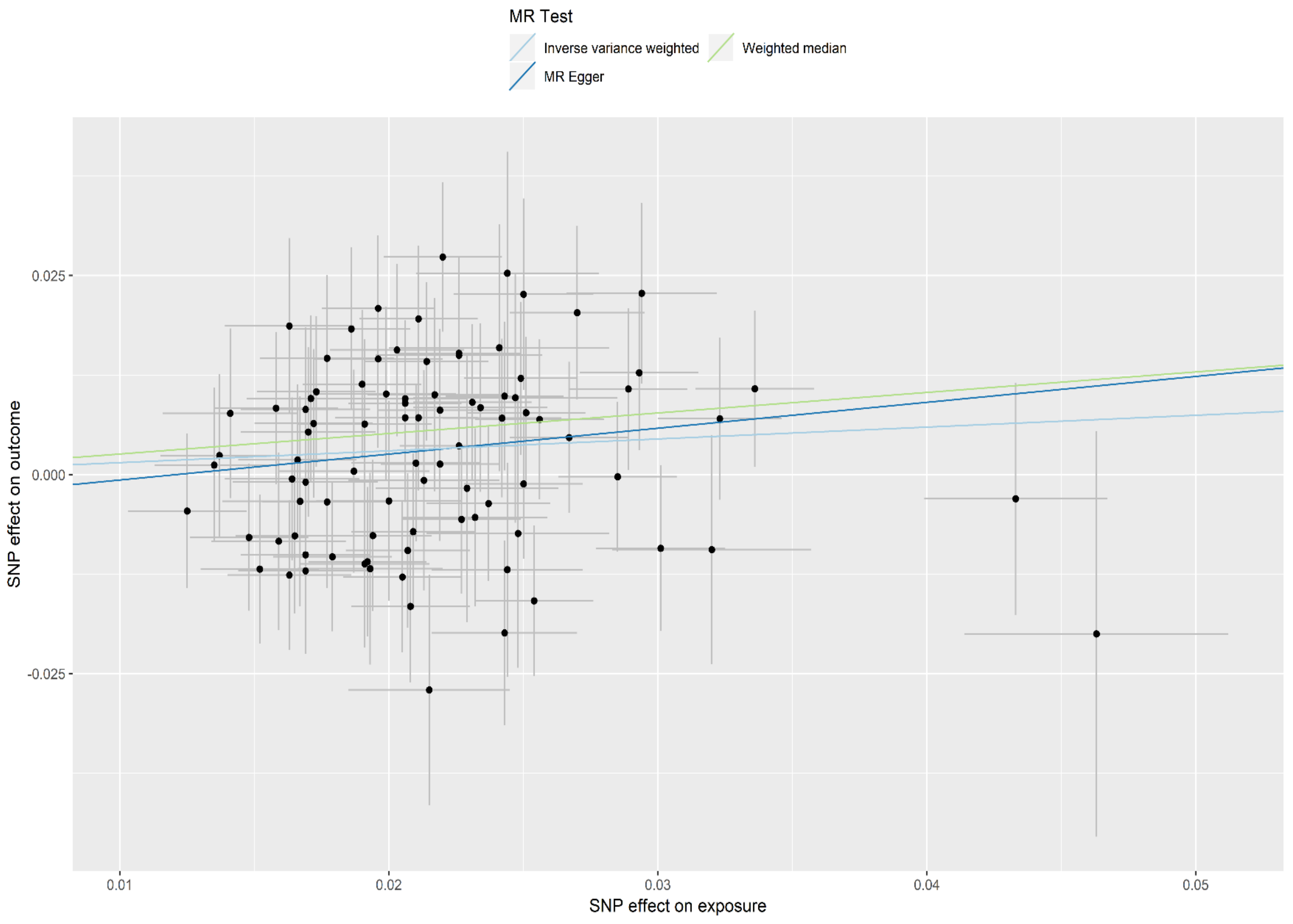

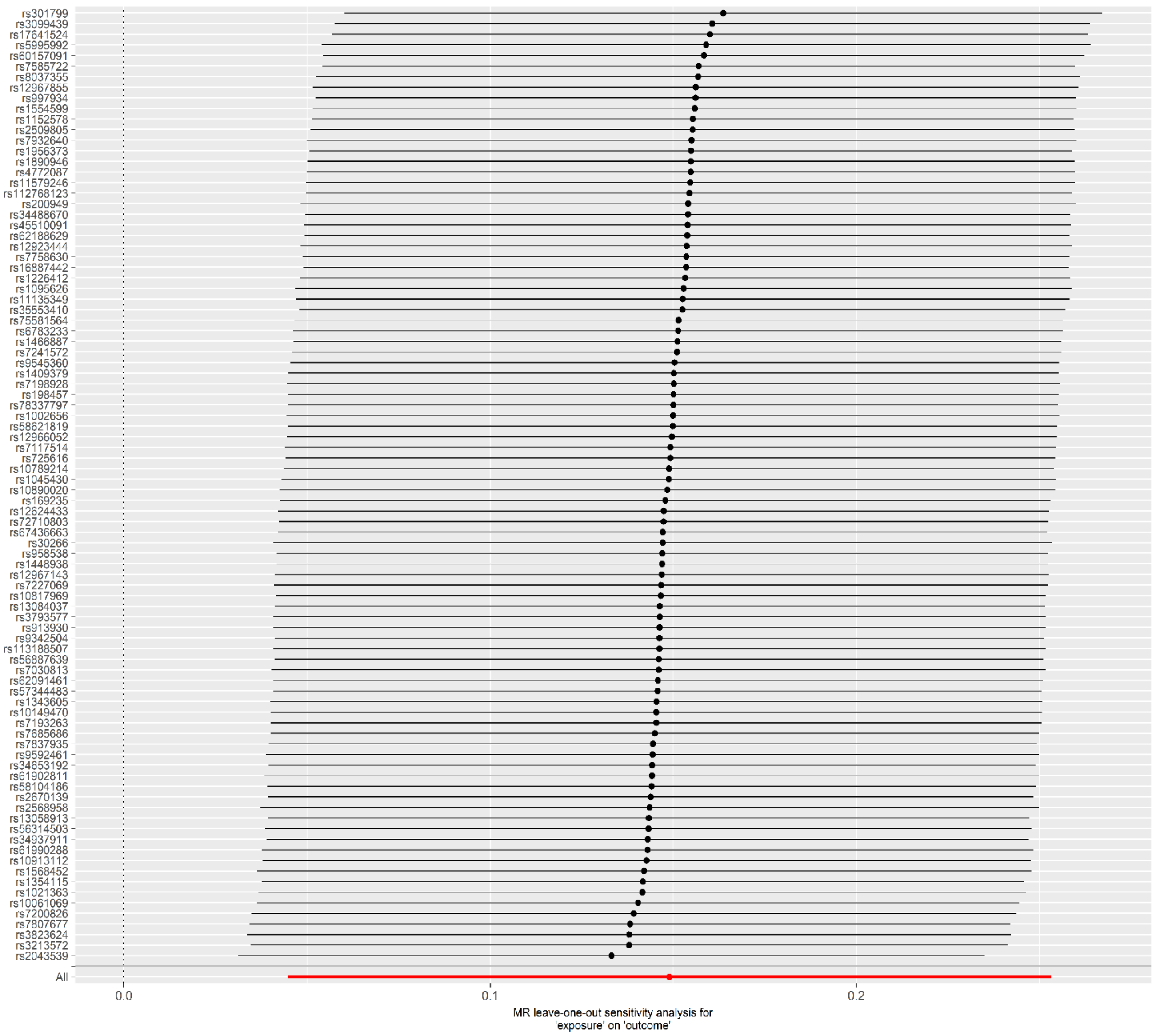

SNP indicates single nucleotide polymorphism; MR, mendelian randomization.

ESM Fig. 4. Scatter plot and leave-one-out analysis for the association of major depressive disorder with heart failure

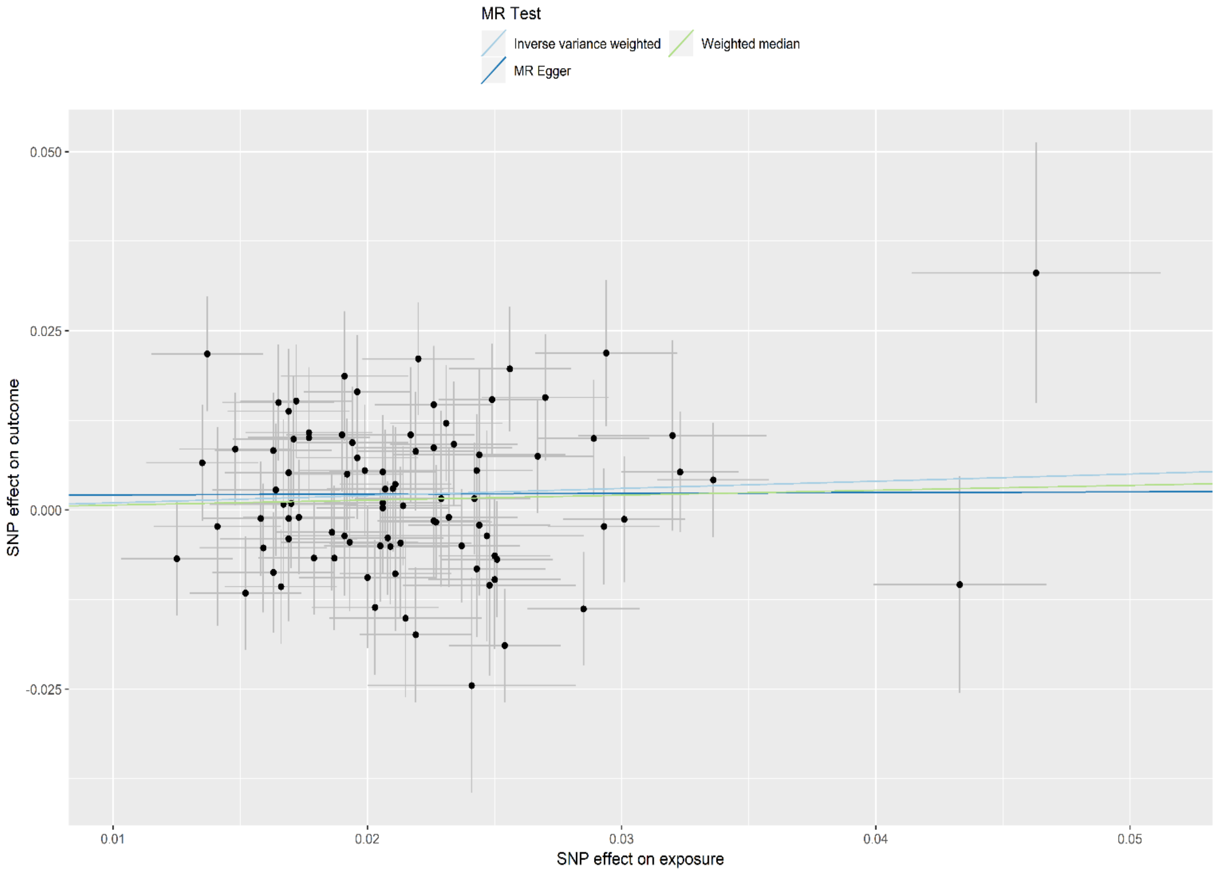

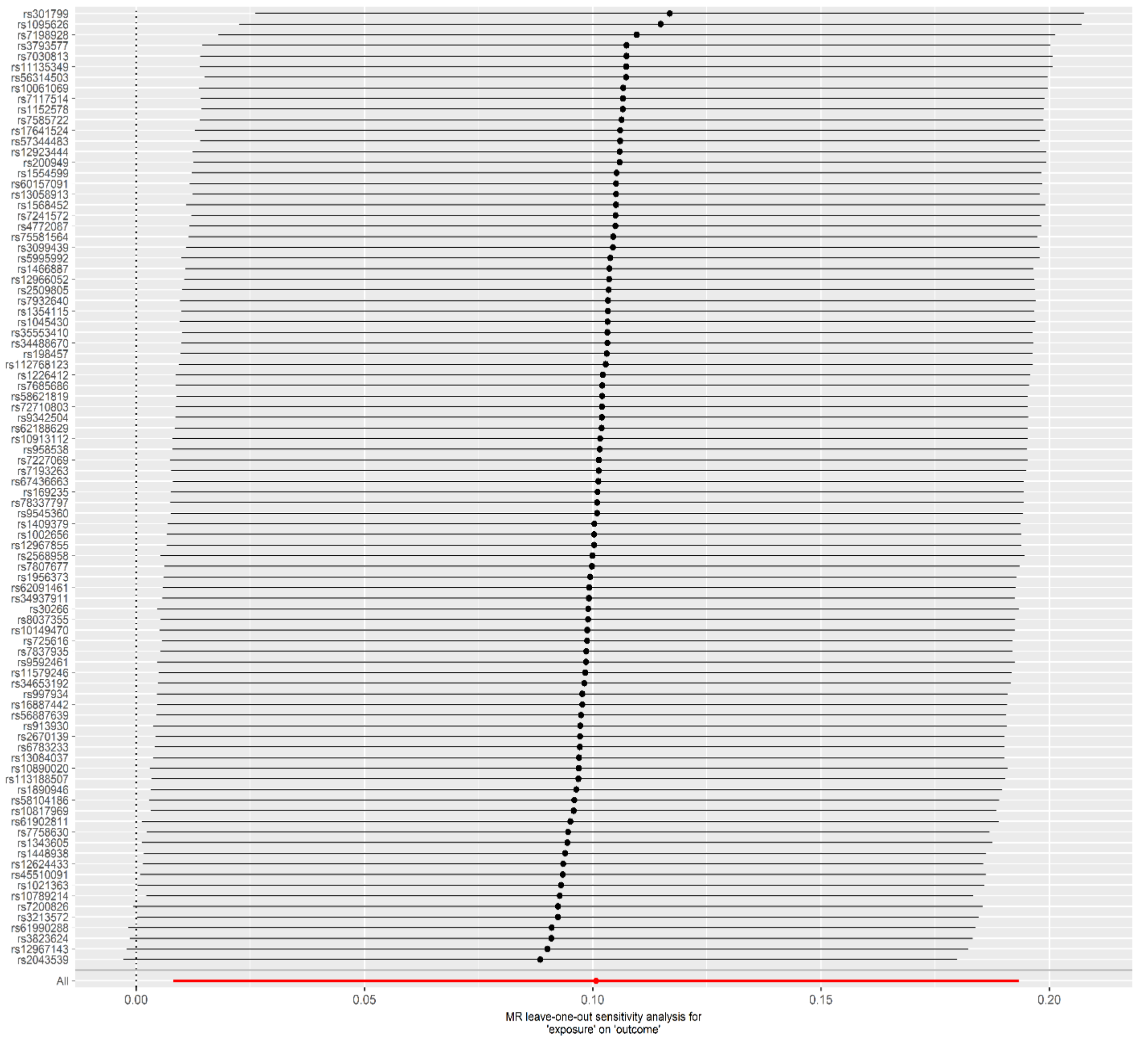

SNP indicates single nucleotide polymorphism; MR, mendelian randomization.

ESM Fig. 5: The association of type 2 diabetes with major depressive disorder using SNPs for type 2 diabetes with and without adjustment for BMI

Exposure	No. of SNPs	Method	OR (95% CI)	p
Type 2 diabetes unadjusted for BMI	344*	Random-effects IVW (Q=616, P<0.001)	1.00 (0.97-1.03)	0.969
		Weighted median	0.99 (0.96-1.03)	0.616
		MR-Egger (P for intercept=0.076)	0.96 (0.90-1.01)	0.117
Type 2 diabetes adjusted for BMI	172**	Random-effects IVW (Q=259, P<0.001)	1.00 (0.98-1.01)	0.487
		Weighted median	0.99 (0.98-1.01)	0.441
		MR-Egger (P for intercept=0.006)	0.97 (0.94-0.99)	0.006
Type 2 diabetes adjusted for BMI	344*	Random-effects IVW (Q=616, P<0.001)	1.00 (0.99-1.01)	0.620
		Weighted median	0.99 (0.98-1.01)	0.300
		MR-Egger (P for intercept=0.022)	0.98 (0.96-1.00)	0.029

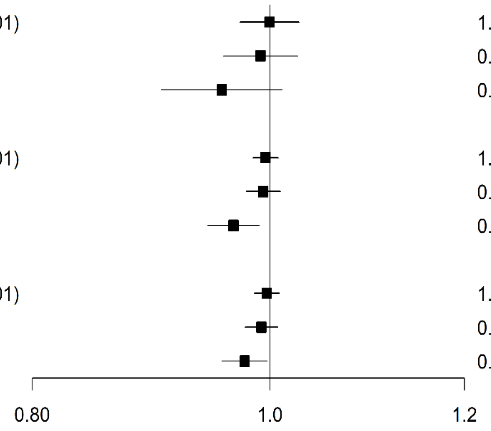

CI indicates confidence interval; IVW, inverse-variance weighted; OR, odds ratio; BMI, body mass index.

* All 403 distinct association SNPs were included in analysis.

** 172 SNPs out of 403 SNPs meeting a genome-wide significant level ($p<5\times10^{-8}$) were included in analysis.
